# Genome-wide binding potential and regulatory activity of the glucocorticoid receptor’s monomeric and dimeric forms

**DOI:** 10.1038/s41467-021-22234-9

**Published:** 2021-03-31

**Authors:** Thomas A. Johnson, Ville Paakinaho, Sohyoung Kim, Gordon L. Hager, Diego M. Presman

**Affiliations:** 1 Laboratory of Receptor Biology and Gene Expression, National Cancer Institute, NIH, Building 41, 41 Library Drive, Bethesda, MD USA; 2 Institute of Biomedicine, University of Eastern Finland, Kuopio, Kuopio, Finland; 3 Instituto de Fisiología, Biología Molecular y Neurociencias (IFIBYNE-UBA-CONICET), Universidad de Buenos Aires, Facultad de Ciencias Exactas y Naturales, Buenos Aires, Argentina

**Keywords:** Steroid hormones, Chromatin immunoprecipitation, Genome-wide analysis of gene expression, Hormone receptors, Transcriptional regulatory elements

## Abstract

A widely regarded model for glucocorticoid receptor (GR) action postulates that dimeric binding to DNA regulates unfavorable metabolic pathways while monomeric receptor binding promotes repressive gene responses related to its anti-inflammatory effects. This model has been built upon the characterization of the GRdim mutant, reported to be incapable of DNA binding and dimerization. Although quantitative live-cell imaging data shows GRdim as mostly dimeric, genomic studies based on recovery of enriched half-site response elements suggest monomeric engagement on DNA. Here, we perform genome-wide studies on GRdim and a constitutively monomeric mutant. Our results show that impairing dimerization affects binding even to open chromatin. We also find that GRdim does not exclusively bind half-response elements. Our results do not support a physiological role for monomeric GR and are consistent with a common mode of receptor binding via higher order structures that drives both the activating and repressive actions of glucocorticoids.

## Abstract (web summary)

Glucocorticoid receptors (GR) are thought to bind DNA as dimers or monomers, to regulate different transcription pathways. Here, the authors perform genome-wide studies on GRs with mutations that impair dimerization and provide evidence that monomeric GRs do not play a significant physiologic role.

## Introduction

The glucocorticoid receptor (GR) is a commonly expressed transcription factor, involved in several aspects of mammalian physiology ranging from development to general homeostasis^1^. It remains inactive in the cytoplasm until hormone binding promotes its nuclear translocation, allowing the receptor to engage with chromatin and modulate gene expression in virtually every tissue^2^. Despite their pleiotropic effects, glucocorticoids (GCs) are widely used in the clinic as powerful anti-inflammatory and immunosuppressive drugs. Unfortunately, chronic GC treatment carries severe side effects, limiting their true potential in treatment of disease^3^.

The quaternary structure of GR was originally proposed as a homo-tetramer on the basis of electron microscopy particle size estimates^4^. Nevertheless, the receptor was later postulated to modulate gene expression via direct DNA binding as a dimer due to the palindromic nature of the glucocorticoid response element (GRE) consensus motif^5^. With the crystal structure of the DNA-binding domain (DBD) fragment, which showed DNA binding enhanced the dimer stability, the community rapidly adopted the dimeric model for direct GR binding. However, the full-length protein was not evaluated in the in vitro study^6^. The region of the DBD critical to dimerization and subsequent transactivation was further narrowed to a segment of five amino acids termed the D-loop^7^.

Other early studies indicated that GR could repress gene activity by tethering to other transcriptional activators such as AP-1 or NFκB^8,9^. In 1994, Cato and colleagues proposed that the activating properties of GR via direct dimeric binding were distinct and separable from its gene repressing properties, where binding occurs as a monomer along with protein partners^10^. Heck et al. further showed that a point mutation (A465T in mouse) in the D-loop generated a monomeric, DNA-binding impaired receptor that could repress an AP-1 dependent reporter but could not activate a GRE reporter. Phenotypic characterization of this mutation in *knock-in* mice (known as GRdim for “dimerization mutant”)^11^ was paramount to the development of the *dissociated model* of glucocorticoid action^12^.

The dissociated model relies on two premises^12^: (1) The GRdim fully supports anti-inflammatory responses to GCs but does not mediate GC-induced side effects. (2) The GRdim cannot form dimers and is unable to directly bind glucocorticoid response elements (GREs) on DNA, abrogating GR’s ability to *transactivate* genes. Hence, it can only bind indirectly to DNA by interacting with and/or inhibiting other transcription factors such as NFκB and AP-1 (i.e., *transrepression*). Consequently, ligands that would allow wild-type GR (GRwt) to mimic GRdim properties should have the same dissociated properties. The search for safer GCs has relied heavily on this model, wherein the oligomeric state of GR defines the transcriptional outcome of the receptor^12^. Unfortunately, research on GR ligands with the goal of shifting the equilibrium towards a monomeric form of the receptor has not been successful.

Later characterization of the GRdim phenotype in transgenic mice has revealed that transrepression and transactivation cannot be as distinctly separated, as previously proposed^13^. Surprisingly, the original characterization of the GRdim did not directly test its oligomeric state^10^; but rather inferred it from a similar mutation on the DBD fragment, in vitro, by EMSA^7^. In addition, a second dimerization surface in the ligand binding domain (LBD) was also reported^14^, questioning the exclusive role of the DBD in GR’s dimerization. We have previously shown by the microscopy technique Number & Brightness that liganded full-length GRdim is mostly dimeric in live cells^15^, consistent with results from another group^16^. However, controversy remains about the extent of GRdim dimer formation, as some partial impairment was reported^17,18^. Nevertheless, we have also demonstrated that full-length GR dimerization can be mostly abrogated via mutations in both the DBD and LBD domains, known as the GRmon^15,19,20^, and when activated by hormone, GRwt exhibits no detectable monomers in the nuclear population^15,21^. Furthermore, receptor binding on an activated GRE in live cells induces higher quaternary structures, suggesting that tetramers may be the final active form of GR^19^. Finally, we have recently shown that a constitutive tetrameric GR mutant is both a better activator and repressor of genes across the genome^22^.

In this work, we engineered a GR null cell system where we reintroduce GRwt, GRdim or GRmon mutants to test the genome-wide role of receptor oligomerization status on GR function. We show that GRdim is indeed a crippled transcriptional modulator, but in ways counterintuitive to predicted modes of action from the dissociated model. GRdim regulates only 20% of the genes controlled by GRwt, and does so less well, even as a transcriptional repressor. The GRmon is almost completely nonfunctional as both a gene activator and repressor. GRdim binds a large subset of GRwt binding sites almost as well on pre-accessible chromatin, including full GREs. In fact, GRwt binding sites that lack chromatin accessibility prior to hormone cannot be opened by GRdim. On the other hand, GRmon binds poorly, even to pre-accessible chromatin. We propose that monomeric GR is not a relevant physiological player and that dimeric or higher oligomeric states constitutes the active form of the receptor.

## Results

### Generation of a GR knock-out/GR mutant cell system

**Fig. 1:**
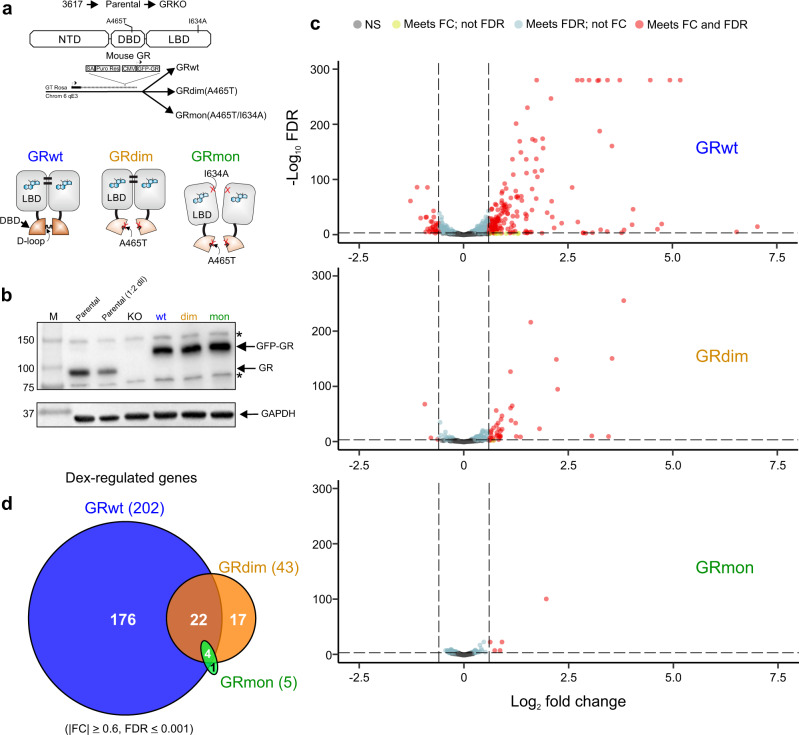
GRdim and GRmon display attenuated transcriptional regulation compared to GRwt. **a** The parental and GRKO cell lines were derived from a C127 murine cell line (3617). The parental cell line contains endogenous GR while the GRKO has endogenous GR knocked out via CRISPR-Cas9. The Cartoon represents the GR mutants used in this study. The wild-type (GRwt) dimerizes through at least two surfaces, located in the ligand binding domain (LBD) and the Distal (D) loop within the DNA-binding domain (DBD). GRdim has a point mutation (A465T) which affects DBD-DBD contacts but remains dimeric through LBD-LBD interactions. GRmon has the A465T mutation as well as an LBD mutation and is therefore monomeric. The GRwt, GRdim, or GRmon of mouse GFP-GR were re-introduced separately into the GRKO cell line and constitutively expressed. **b** Western blots for GR. GAPDH is a loading control. M, molecular weight marker. Endogenous GR, GFP-GR and GAPDH bands are highlighted on the right with arrows; * denotes non-specific bands. GAPDH blot was repeated on a separate gel due to an air bubble during protein transfer (Supplementary Fig. 9). The absence of a GR band in the KO cell line was independently confirmed three times, while the expression levels of GR mutants were done twice. **c** Volcano-plot representation from RNA-seq data after 2 h Dex treatment. NS, non-significant. FC, fold change. FDR, false discovery rate. The dashed lines indicate the thresholds used (|FC|> 0.6, FDR < 0.001) and the different colors in each datapoint indicate whether they meet FC, FDR, or both criteria. **d** Venn diagram of differentially expressed genes from RNA-seq data after 2 h Dex treatment.

We created a GR knock-out (GRKO) cell line from a mouse mammary adenocarcinoma cells with no detectable receptor nor hormone response^22^. We used this cell line to stably reintroduce GFP-tagged wild-type or mutant forms of GR to study receptor function and gene response. The three new cell lines express either GRwt, the GRdim (A465T) or the GRmon (A465T/I634A) mutants (Fig. 1a) at similar levels to endogenous GR in the parental line (Fig. 1b). We collected genome-wide data sets for RNA, chromatin immunoprecipitation (ChIP), and chromatin accessibility in the cell lines before and after hormone treatment to compare the respective function of each receptor type.

### A monomeric GR is insufficient to elicit a gene response

A functional correlation between the oligomeric state of GR and transcriptional modulation has been a central thesis for the dissociated model of GC action^12^. We performed RNA-seq on at least two biological replicates of each cell line for the untreated condition and three replicates each for the hormone treatment condition. We performed differential expression (DE) analysis while accounting for the unwanted variation in the RNA-seq datasets from each cell line using remove unwanted variation from RNA-Seq data (RUVSeq) method^23^. We chose a stringent false discovery rate (FDR) cutoff of 0.001, and a log_2_ fold change (FC) ≥ 0.6 as determined by DESeq2^24^ in order to be confident that the genes are not only differentially regulated, but show a substantial (more than 1.5 fold) difference in expression.

Comparing RNA-seq data before and after 2 h dexamethasone (Dex) (100 nM), we identify 202 hormone-regulated genes in the GRwt cells compared to only 43 and 5 regulated genes in the GRdim and GRmon cells, respectively (Fig. 1c, d). Around half of GRdim regulated genes (26 out of 43) overlap with GRwt genes. Of the five GRmon regulated genes, four of them are shared with GRwt and GRdim (Fig. 1d; Supplementary Data 1). Of note, GRdim and GRmon are particularly deficient at down-regulation, with only three genes for GRdim and none for GRmon, compared to 32 genes by GRwt (Fig. 1c and Supplementary Fig. 1a). Using a less stringent FDR cutoff of 0.05 only modestly increases the number of GRwt (212) and GRdim (45) hormone responsive genes, while no additional changes occur with GRmon. Removing the 0.6 log_2_ FC requirement (with FDR cutoff of 0.001) still shows an attenuated response to hormone in GRdim cells and even more so in GRmon cells (Fig. 1c and Supplementary Fig. 1c).

Assessment of the basal (non-treated) expression levels among a common set of 13,465 expressed genes by Principal Component Analysis (PCA) revealed some differences between each mutant cell line, even though they still clustered together (Supplementary Fig. 2a). A similar effect has been observed on a recent report comparing GRwt and a DBD binding mutant in primary mouse embryonic fibroblast cells^25^. A closer inspection revealed that only 43 genes (out of 13,465) are differentially regulated (using a 0.6 log_2_ FC and 0.001 FDR) between GRwt and GRmon backgrounds, with only 5 genes overlapping with hormone-dependent action. Background level differences were less pronounced on GRdim, as we detected only one differentially expressed gene compared to GRwt. One possibility is that GR reintroduction produces some divergence in the cell lines background levels by either ligand-independent or -dependent mechanisms during clonal expansion and cell growth. Alternatively, genetic drift after transgene insertion is also plausible. Nonetheless, the baseline differences detected by PCA does not appear to affect the hormone response of the three cell lines. We plotted the normalized RNA read counts for the union of hormone-regulated genes (220) across the cell lines (Supplementary Fig. 2b). The no-treatment scatter plots of hormone responsive genes show little variability between biological replicates of GRwt, GRdim and GRmon, with R-squared values of 0.95 or higher. Indeed, we observe much larger differences in RNA levels from the hormone treatment in GRwt compared to GRdim and to GRmon (Supplementary Fig. 2c). Moreover, the basal chromatin accessibility as measured by ATAC-seq was nearly identical between GRwt- and GRdim-expressing cells (Supplementary Fig. 2d), suggesting isogeneity at the chromatin landscape level. Lastly, even if we take into account baseline differences across cell lines (see methods for details), we consistently observe a wide scale attenuated gene response with GRdim (Supplementary Fig. 3a, b), and more so with GRmon (Supplementary Fig. 3c, d).

Taken together, the lack of a response in GRmon after hormone activation is therefore likely due to its functionality and not to differences between the cell lines used in this study. Even though our engineered cell lines are not fully isogenic, they remain a suitable model to study the effect of each mutant within their own genetic backgrounds.

To determine if the GRdim and/or GRmon merely delay the response observed by GRwt, we performed by reverse transcriptase (RT)-qPCR a hormone response time-course of several Dex up-regulated genes. Even after 6 h of hormone treatment, the transcriptional response as measured by nascent RNA levels is severely attenuated by both GRdim and GRmon (Supplementary Fig. 4). In conclusion, a forced monomeric form of GR appears unable to elicit a robust transcriptional response.

### A monomeric GR binds poorly to chromatin

It has been argued that the GRdim mutant is not able to bind DNA;^11^ however, several reports do not concur with this finding^15,16,26,27^. Furthermore, controversy remains regarding the role of GR’s oligomeric state in predicting chromatin binding^28^. We therefore performed ChIP-seq for cell lines expressing each of the mutants before and after 1 h Dex (100 nM). We used a GFP antibody as it correlates well with a cocktail of anti-GR ChIP-seq data but provides a stronger signal^22^.

**Fig. 2:**
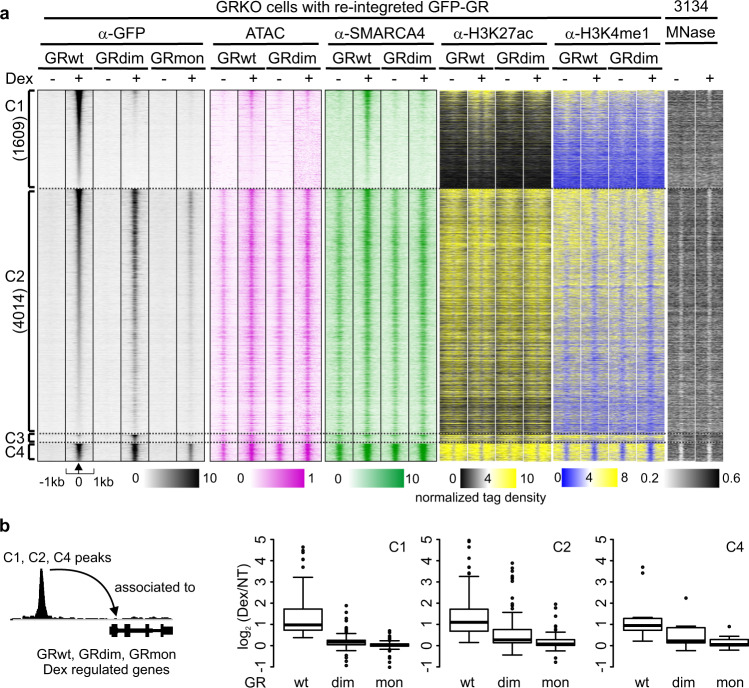
Chromatin binding of GRwt, GRdim and GRmon. **a** Comparison of GRwt, GRdim, and GRmon binding reveals four clusters, C1–C4; C1 GRwt specific; C2 shared by GRwt and GRdim; C3 GRdim specific; C4 shared by all three. Heat maps represent ChIP-seq, ATAC-seq, MNase-seq data as indicated. Each heat map represents ±1 kb around the center of the GR peak. Treatment indicated on top; binding intensity scale noted below on a linear scale. Heat maps sorted based on GRwt binding intensity, except C3 which is sorted on GRdim binding intensity. All heat maps normalized to 10 million reads, and further to local tag density. **b** C1, C2 and C4 sites are associated with nearest Dex up-regulated gene based on linear proximity. The up-regulated genes are from a composite list from the three cell lines, *n* = 195 genes. Box plots represent the overall log_2_ fold change (Dex/NT) of genes in the respective cell line to called ChIP peaks from each cluster. No association of Dex up-regulated genes observed with C3 peaks. Box plots were generated with Tukey method with interquartile range (IQR) depicting the 25th, 50th and 75th percentile as box with the median as black bar. The whiskers extend 1.5xIQR beyond the box, and outliers depicted as circles extend beyond the 1.5xIQR. Source data are provided as a Source Data file.

**Fig. 3:**
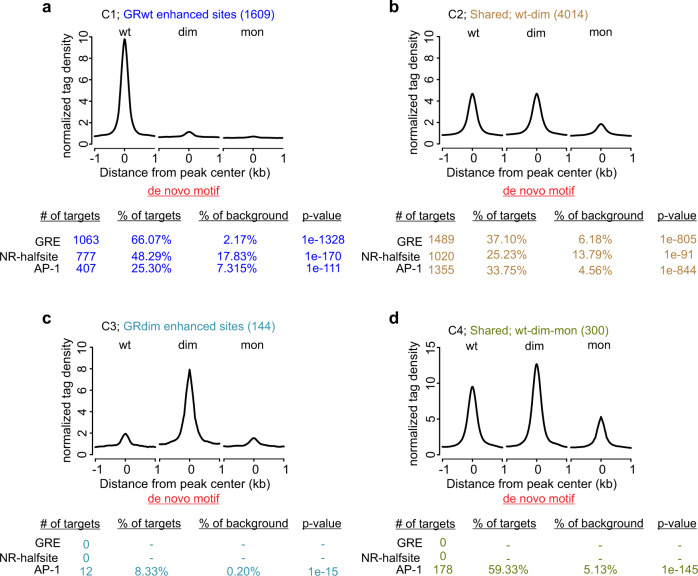
Chromatin binding and de novo motif analyses of GRwt, GRdim, and GRmon. **a**–**d** Aggregate plots of +Dex GR binding (α-GFP) of each receptor type for each cluster. All aggregate plots normalized to 10 million reads, and further to local tag density (tags per site per bp). De novo motif analysis shows the percentage of sites with a motif and the calculated background percentage from 50 K random sites (with matched GC% content). p-value of the motif enrichment is shown. Source data are provided as a Source Data file.

Cluster analyses of the GFP-GR binding profiles of GRwt, GRdim, and GRmon reveal four distinct groups comprising a total of 6067 binding sites across the three cell lines (clusters C1–C4, Figs. 2a, 3). None of these peaks are observed in the GR knock-out cell line (Supplementary Fig. 5). ChIP peaks are aligned on their GR peak summits, and sorted to obtain a heat map with the most highly occupied sites at the top of each cluster (Fig. 2a). C1 represents 1609 sites (27%) that are enriched significantly more by GRwt than by either mutant receptor. The largest cluster, C2, comprises 4014 sites (66%) occupied equally by both GRwt and GRdim but not GRmon. Even though the heatmap appears to show some positive signal for GRmon at this cluster (Fig. 2a), an aggregate plot reveals very low enrichment (Fig. 3b, Supplementary Fig. 6a). C3 consists of only 144 sites (2%) where GRdim binds exclusively. The C4 cluster includes 300 sites (5%) where all three receptor types bind and is the only cluster where GRmon meets the criteria for called peaks (see Methods), albeit at lower enrichment than GRwt or GRdim (Fig. 2a, Supplementary Fig. 6a). It should be noted that the small cluster of C3 sites are occupied by the endogenous GR in the 3134 cell line^29^ (Supplementary Fig. 6b), indicating that the lack of GRwt binding may be related to a slightly reduced activity for the GFP-tagged transgene receptor. The clear majority (>90%) of GR binding sites occur within distal intergenic and intronic genomic regions except C3, where 25% of binding sites occur at promoters and other miscellaneous sites (Supplementary Fig. 4c). Representative genome browser track examples are shown in Supplementary Fig. 6d–g.

The low level of GRmon binding correlates with its poor hormone response via RNA-seq (Fig. 1c). Alternatively, the diminished gene regulatory activity of GRdim, as indicated by the RNA-seq data (Fig. 1c), stands in contrast to its ability to bind chromatin at over 70% of sites (C2 and C4) bound by GRwt (C1, C2 and C4) (Fig. 2a). Determining the proximity of Dex-regulated genes in GRwt, GRdim, or GRmon expressing cells for C1 sites indicates GRwt binding is associated with up-regulation of the nearest hormone induced genes but this association is lost in GRdim and GRmon cells (Fig. 2b). Dex up-regulated genes associated with GR binding sites in C2 are weakly regulated in GRdim cells compared to GRwt, while the association is absent in GRmon cells. This trend is also observed for the three cell lines with C4 sites. Furthermore, the GRdim-specific C3 sites showed no association to Dex-regulated genes. Taken together, our data indicate that a monomeric GR is unable to regulate genes because it binds poorly to chromatin.

### GRdim does not bind primarily to half-GREs

Although our previous work demonstrated the GRdim is mostly dimeric in live cells^15^, evidence for monomeric binding has been presented in the form of half-GRE motifs recovered from de novo motif analysis in ChIP studies^26,27^. Similar analysis on our data (Fig. 3a, b, Supplementary Data 2) shows full GRE and half-GRE enrichment at C1 (GRwt enhanced) and C2 (shared GRwt/GRdim), but not at C3 (GRdim enhanced) or C4 (Shared GRwt/dim/mon) (Fig. 3c, d). Thus, based on de novo motif enrichment, GRwt and GRdim can bind to both full and half sites, while GRmon appears unable to bind either. The inability of GRmon to bind half-GREs suggests that both GRdim and GRwt binding to these sites is unlikely to occur as monomers. Consistently, we reported that GR dimers can be held independent of DNA by LBD-LBD interactions^15,19^. Therefore, it is possible that GR dimers can “land” on half-GREs and remain dimeric.

**Fig. 4:**
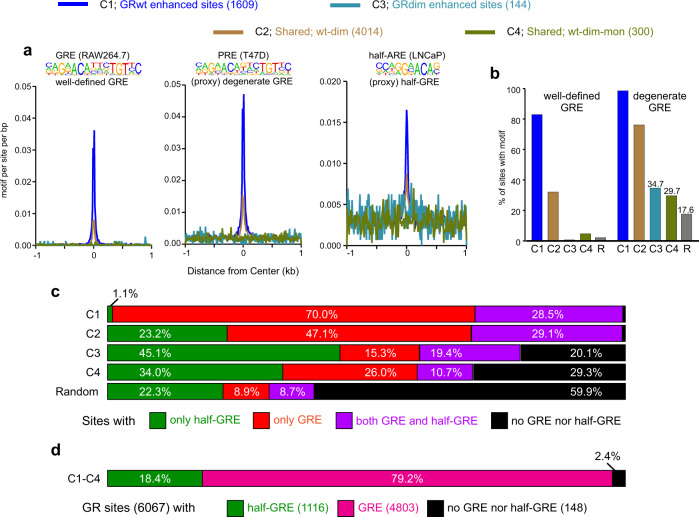
Pre-defined motif analyses. **a** Motif enrichment of a well-defined GRE (HOMER motif gre-raw.motif), a proxy for a more degenerate GRE (HOMER motif pr.motif) and a proxy for a half-GRE (HOMER motif ar-half.motif) at GR ChIP peaks from each cluster (C1–C4). Position-weight matrix (PWM) logo for each GRE shown above each aggregate plot. All aggregate plots normalized to 10 million reads, and to motif per site per bp. **b** The percentage of sites within each cluster (C) that contain each pre-defined motif as well as the percentage at 2000 randomly chosen (R) ATAC peaks from the Dex-treated GRwt cell line. **c** The percentage of GR peaks with only a full GRE (red color), only a half-GRE (green color), both a full GRE and half-GRE (purple color), or no GRE nor half-GRE (black color). The degenerate full GRE and half-GRE in **a** were used to determine the percentages. **d** The percentage of all GR peaks (6067) that contain half-GRE (green), full GRE (magenta) or no half nor full GRE (black). Source data are provided as a Source Data file.

Even though the GRE consensus motif does not occur often enough to be detected by the de novo analysis at C3 and C4, the C4 group harbors the well-known Per1 regulatory site that contains a strong full GRE motif (Supplementary Fig. 6g). Due to a potential miscall from the de novo analysis, we performed pre-defined motif analyses to detect occurrences of GREs more efficiently (Supplementary Data 3 and see methods for details). For a well-defined GRE, we chose the RAW264.7 GRE position weight matrices (PWMs) from HOMER^30^ as it is generated from mouse cells (Supplementary Fig. 7a). As a proxy for a degenerate GRE, we chose PR’s PWMs motif from HOMER, as it represents a “more relaxed” form of a GRE by having a less stringent motif at the ACA and TGT core base pairs (Supplementary Fig. 7a). Finally, for half-GRE, we chose HOMER’s AR half site motif as it has been previously used to define GRdim binding to half-GREs^26^. We plotted the relative enrichment of these GREs at the GR ChIP peak summits (Fig. 4a). All GREs are more enriched at C1 sites than C2 sites with very little enrichment at C3 and C4 sites, consistent with the de novo analysis. We further did pre-defined motif analyses with all NR3C type motifs available in HOMER and all mouse GRE PWMs found at CIS-BP database^31^. All the different GRE PWMs show similar results, wherein full GREs are enriched at C1, less so at C2 and not at C3 and C4 sites (Supplementary Fig. 7a, b). As evidence is emerging that GR and AR might not have the same preference for SREs^32^, we also include a half-GRE from CIS-BP (instead of a half-ARE) for pre-defined motif analysis, obtaining similar results (*c.f*. Fig. 4a and Supplementary Fig. 7b). The strength of the enrichments varies with the stringency of eight called bases of the GR motif. Log-odds motif scoring (see Methods for details) agrees with the motif enrichment plots (Supplementary Fig. 7c). We then calculated the percentage of the GR sites within each cluster that contain a well-defined or degenerate GRE (as defined in Fig. 4a) within the breadth of the GR ChIP peak, as well as the percentage within 2000 randomly selected accessible sites (Fig. 4b). In contrast to the de novo analysis for C3 and C4 sites (no detected GREs), these clusters have slight enrichments of the degenerate full GREs (C3; 34.7% and C4; 29.7%) over random accessible sites (17.6%) (Fig. 4b). This indicates some level of full GRE binding, but also likely means that some sites have GR binding via half-GREs, non-specific binding or binding via association with another factor. To further address this point, we determined the percentage of sites in each cluster that contain only a full GRE (well-defined or degenerate), only a half-GRE, both a full and half-GRE, or none (Fig. 4c; see Methods for details). Almost all C1 sites (~98%) contain a full GRE or both a full and half-GRE with only 1.1% containing only a half-GRE. About 75% of C2 sites (red + purple, Fig. 4c) contain a full GRE or both a full and half-GRE while 23% contain only a half-GRE, about the same as the 2000 randomly selected accessible sites (22.3%). C3 and C4 sites have somewhat more half-GREs than random accessible sites, 45% and 34%, respectively and about 35% of full GREs with or without an additional half-GRE. Overall, 79% of the 6067 GR bound sites have a full GRE while 2.4% have no full or half-GRE (Fig. 4d). The remaining 18.4% of GR sites, all of which are in C2–C4, solely contain a strong consensus half-GRE. We cannot rule out that even some portion of these sites are full GREs that are more degenerate than the motif used as a proxy in our pre-defined search versus all being genuine half-GREs. For example, the even more degenerate GRE motif ACAnnnTnT occurs in 26 out of the 65 C3 half GREs and 36 out of the 102 C4 half GREs defined in Fig. 4a. Subtracting these sites from the C3 and C4 half GREs lowers the percentage of sites with a half GRE to 27 and 22%, respectively. This is similar to the percentage of half GREs in random sites (22.3%), as shown in Fig. 4c. Collectively, the motif analyses largely weaken the argument that monomeric GR is an important contributor to the overall functionality of GR, as concluded by Cohen and colleagues^33^. While we cannot rule out the possibility that GR can bind chromatin as a monomer in the nucleus of the cell, no solid evidence exists to argue that it does.

**Fig. 5:**
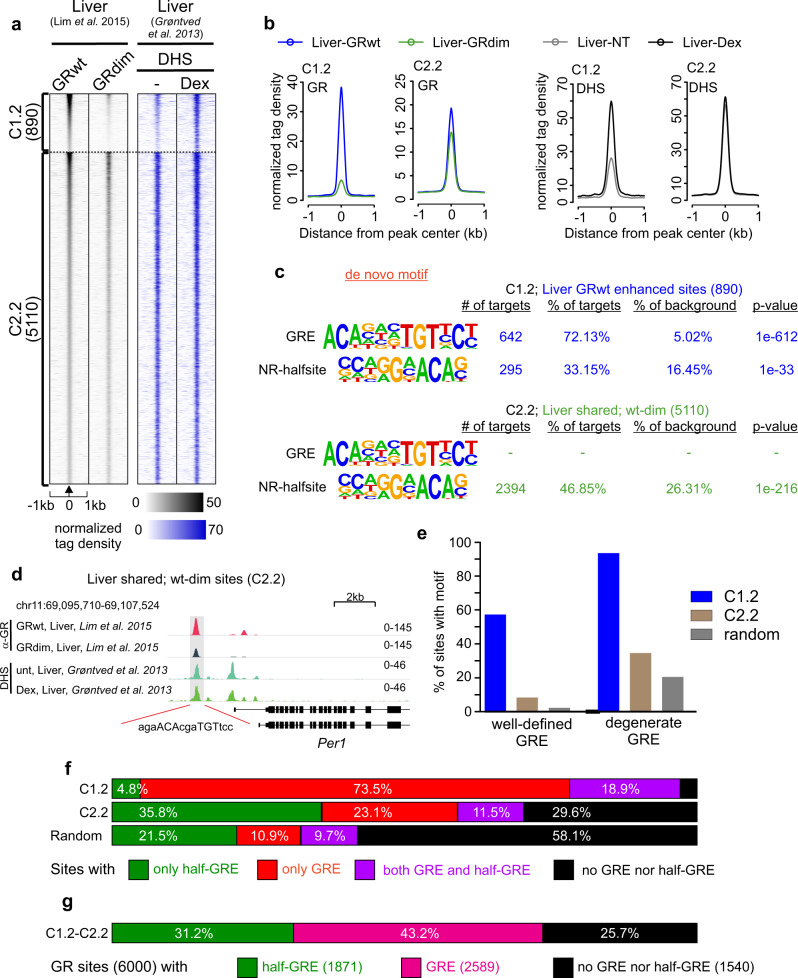
Liver GR chromatin binding and de novo motif analyses. **a** Comparison of GRwt and GRdim ChIP-seq data (black) in mouse liver reveals two clusters, C1.2 (GRwt specific) and C2.2 (GRwt/GRdim shared). DNase I hypersensitive sites (DHS) (blue) from mouse liver also shown. Each heat map represents ±1 kb around the center of the GR peak. Binding intensity scale noted below on a linear scale and treatment noted above for DHS. Heat maps sorted based on GRwt binding intensity and normalized to 10 million reads and to local tag density. **b** Aggregate plots of GRwt (blue) and GRdim (green) binding for each cluster and the corresponding DHS (GRwt-black, GRdim-gray). All aggregate plots normalized to 10 million reads, and further to local tag density (tags per site per bp). **c** De novo motif analysis shows the percentage of sites with a motif and the calculated background percentage from 50 K random sites (with matched GC% content), p-value of the motif enrichment. The PWM logo of detected motif shown on left. **d** Genome browser track of Per1 locus with perfect GRE (gray highlight) and GR binding sites in each cell line. Tracks are normalized to total of 10 million reads. **e** The percentage of sites within each cluster that contain each pre-defined motif (see Fig. 4b) as well as the percentage at 2000 randomly chosen DNase-seq peaks from the mouse liver. **f** The percentage of GR peaks with only a full GRE (red color), only a half-GRE (green color), both a full GRE and half-GRE (purple color), or no GRE nor half-GRE (black color). The degenerate full GRE and half-GRE in Fig. 3a were used to determine the percentages. **g** The percentage of all GR peaks (6000) that contain half-GRE (green), full GRE (magenta) or no half nor full GRE (black). Source data are provided as a Source Data file.

The conclusions of our motif analyses stand in contrast to liver data reported from GRdim *knock-in* mice^26^, where it was shown that sites shared between GRwt and GRdim are enriched only with half-GRE motifs and devoid of full GREs. These results were interpreted as monomeric engagement of GRdim to chromatin^33^. We re-analyzed these GRwt and GRdim liver datasets and found they clustered similarly to our cell lines; C1.2 represents sites occupied significantly more by the GRwt than GRdim, and C2.2 are shared by both receptors (Fig. 5a, b). Our de novo motif analyses, in agreement with Lim et al., show full GRE motifs enriched only at C1.2 sites, whereas half-GRE motifs are enriched at both C1.2 and C2.2 (Fig. 5c). The C2.2 group harbors the well-known Per1 regulatory site with a full GRE (Fig. 5d), suggesting misclassification is also apparent in liver data. To evaluate the extent of possible misclassification of half-GREs, we performed the same pre-defined motif analyses as described above with well-defined and more relaxed GRE motifs, and determined the percentage of sites in each cluster that contain only a full GRE, only a half-GRE, both a full and half-GRE, or none (Fig. 5e, f). As in our adenocarcinoma cell lines, the majority of C1.2 motifs are full GREs (Fig. 5e). On the other hand, C2.2 does contain a higher percentage of half-GREs than C1.2 but are only about two-fold enriched over 2000 randomly selected accessible sites (35.8% vs 21.5%, Fig. 5f). In striking contrast to the de novo motif analysis, a noteworthy proportion of motifs in C2.2 are full but degenerate (more relaxed) GREs (Fig. 5e, 34.6%), and some C2.2 are even well-defined (Fig. 5e, 8.4%). Moreover, C2.2 presents a similar percentage of full GREs and half-GREs (green, 35.8% vs red + purple, 34.6%, Fig. 5f). Overall, 43.2% of the 6000 liver GR bound sites have a full GRE, 31.2 % have a half-GRE while 25.7% have no full or half-GRE (Fig. 5g).

Taken together, our results caution against relying solely on HOMER de novo motif analyses to assess global GR binding, as some motifs can be misclassified due to degenerate sequences. For example, full GRE binding was completely missed by the de novo analysis at the shared GRwt/dim sites in liver. Our data make a strong argument that GRdim does not bind predominantly to half-GREs. Therefore, dimeric binding by GRdim cannot be ruled out by this kind of genomic analysis.

### Chromatin pre-accessibility determines GRdim occupancy

Why does the GRdim elicit such a poor transcriptional response compared to GRwt? The two original explanations, inability to form dimers or to bind DNA, are inconsistent with (1) our imaging data indicating GRdim can form higher quaternary structures^19^, and (2) the ChIP-seq data (Fig. 2a), clearly showing chromatin binding. To gain more insight into the different behavior between GRwt and GRdim, we characterized the chromatin environment of receptor bound sites by measuring chromatin accessibility by ATAC-seq, the binding profile of the chromatin remodeler SMARCA4 (also known as BRG1), an important GR cofactor^34^, and the active and poised enhancer status by ChIP-seq of H3K27ac and H3K4me1 marks^35^.

**Fig. 6:**
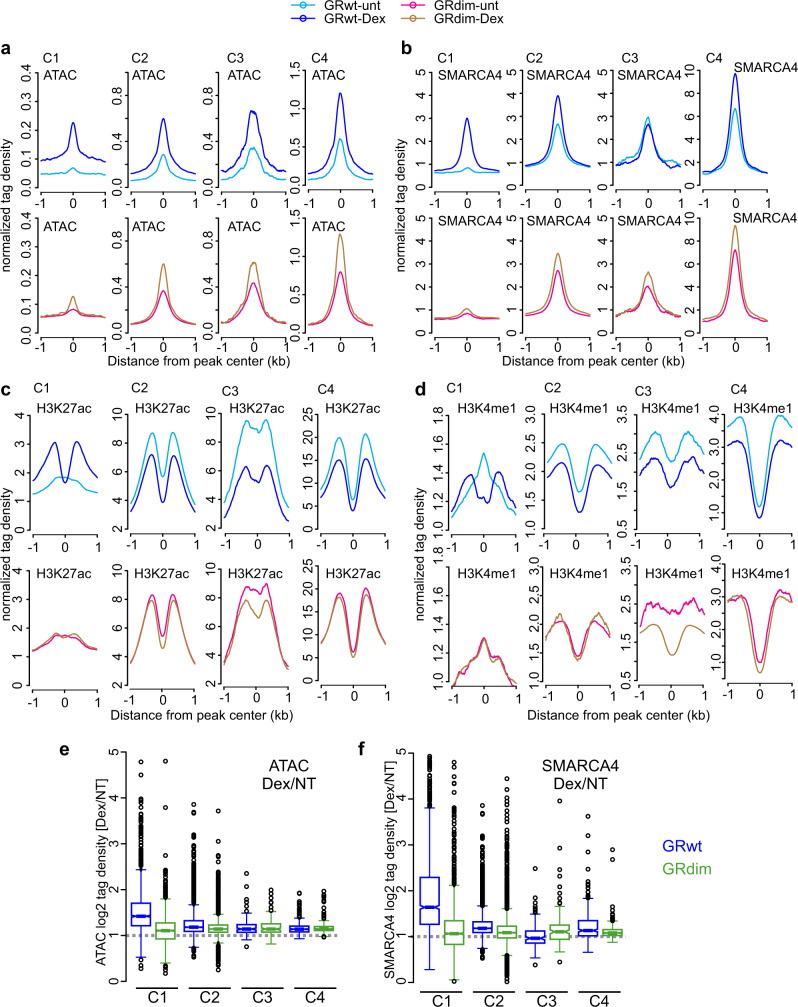
GRwt can bind to closed chromatin sites and increase enhancer accessibility and activity. **a–d** Aggregate plots represent ATAC-seq (**a**), SMARCA4 (**b**), H3K27ac (**c**) and H3K4me1 (**d**). ChIP-seq changes for each cluster; color indicates treatment and GR type. **e**, **f** Box plots represent Dex/NT comparison of normalized log_2_ tag density of ATAC-seq (**e**) and SMARCA4 ChIP-seq (**f**) between of GRwt (blue) and GRdim (green) at C1–C4 sites. C1 *n* = 1609, C2 *n* = 4014, C3 *n* = 144, C4 *n* = 300 sites. All box plots are normalized to total of 10 million reads. Box plots were generated with Tukey method with interquartile range (IQR) depicting the 25th, 50th and 75th percentile as box with the median as black bar. Notches depicting the confidence of the median. The whiskers extend 1.5xIQR beyond the box, and outliers depicted as circles extend beyond the 1.5xIQR. Source data are provided as a Source Data file.

The C1 sites, bound almost exclusively by GRwt, display a clear lack of chromatin accessibility and SMARCA4 prior to hormone while the C2–C4 clusters, where GRdim binds, are pre-accessible and more enriched for SMARCA4 before hormone (Fig. 1d, Supplementary Fig. 6b, Fig. 6a, b). This suggests GRdim can bind only where chromatin is most amenable to receptor access. The histone modification data parallel the chromatin accessibility data by demonstrating that C1 sites, before hormone, are marked by H3K4me1, indicating poised enhancers, whereas the C2–C4 sites are positive for H3K27ac (Fig. 2a, Fig. 6c, d), a mark of active enhancers. Furthermore, nucleosome mapping in the 3134 cell line by MNase-seq^36^ indicates that C2–C4 sites are depleted of nucleosomes prior to hormone, while C1 sites are more nucleosomal (Fig. 2a). After Dex treatment, C1 sites in GRwt cells increase in chromatin accessibility, SMARCA4 enrichment, and GRE-adjacent H3K27ac while hormone dependent changes at this cluster in GRdim cells are lower or absent (Fig. 2a, Fig. 6a–d). Only slight increases in the pre-existing chromatin accessibility and SMARCA4 enrichment occur after Dex in both cell lines at C2 sites, while few observable changes occur at C3 and C4 sites (Fig. 2a and Fig. 6a,b,e,f). The histone acetylase EP300, which is suggested to predict GR chromatin binding^37^, is enriched at the clusters in the same manner as chromatin pre-accessibility or occupancy of SMARCA4 (Supplementary Fig. 5b). In a complementary fashion, the GRwt enhanced sites (C1.2) in liver are less accessible prior hormone treatment (Fig. 5a, b) as indicated by DNase-seq data in liver^38^. Furthermore, these sites become more accessible after Dex treatment whereas hormone has seemingly no effect in the chromatin accessibility of the shared (C2.2) liver sites. These analyses further confirm that GRwt is more capable than GRdim to bind to closed chromatin sites and can more effectively alter the local chromatin environment in a way that is conducive to transcription.

Consistent with the important role AP-1 has on GR binding^39^, de novo motif analyses shows enrichment of the AP-1 motif at all GR binding clusters to varying degrees (Fig. 3a–d) with the GRdim specific C3 having the lowest enrichment. There are no other clear differences in motif enrichment between the clusters (Supplementary Data 2). The enrichment of activated receptor binding by both GRwt and GRdim is correlated with the strength of the GRE motif, where the motif is strongest in C1, weaker in C2, very weak or absent in C3 and C4 (Supplementary Fig. 7c). However, C2 contains almost two-fold higher number of full GRE binding sites (Fig. 4c (47.1% + 29.1% = 3059 peaks)) than C1 (Fig. 4c (70% + 28.5% = 1585 peaks)), suggesting that GRdim binds to full GRE containing sites just as well as GRwt. Hence, chromatin accessibility rather than motif-strength explains better the differential binding between GRwt and GRdim at C1 sites. On the other hand, the motif score of the half-GRE is roughly equal between the clusters (Supplementary Fig. 7c). This further suggests that the half-GRE does not contribute to the binding differences observed between GRwt and GRdim. The AP-1 motif is stronger within the pre-accessible C2 and C4 clusters than the C1 (Supplementary Fig. 8a), suggesting that AP-1 may be at least partially responsible for recruiting factors like SMARCA4 and maintaining the pre-hormone accessibility absent in C1. Indeed, pre-hormone AP-1 binding is present at C2 and C4 sites in the 3134 cell line (Supplementary Fig. 5b), and GR binding is also significantly affected by the expression of a dominant-negative AP-1 at these sites (Supplementary Fig. 8b). Furthermore, the sites at C2–C4 are very close to an AP-1 peak (median 25–120 bp), whereas C1 sites are farther from the AP-1 peak (median 2.7 kb, Supplementary Fig. 8c). Thus, these results suggest that while C1 sites are AP-1 independent, C2 and C4 sites depend on the activity of AP-1.

Taken together, the ChIP-seq and ATAC-seq data sets indicate GRdim is impaired to bind pre-hormone inaccessible chromatin and induce sufficient changes in local chromatin accessibility and histone marks to enable an efficient transcriptional response compared to GRwt. The inability to bind closed chromatin is further illustrated by re-sorting C1 sites high to low based on nucleosome occupancy at the GRE in untreated 3134 cells (Supplementary Fig. 8d). Slight increases in chromatin accessibility with hormone can only be seen for GRdim at C1 GREs with the lowest nucleosome occupancy. Overall, our results suggest that only GRwt can act as an initiating factor by binding to closed nucleosomal chromatin sites and increase chromatin accessibility and enhancer activity.

## Discussion

The quaternary structure of GR is presumed to be of important pharmacological relevance, yet its oligomeric status is still a matter of debate^28,40,41^. The search for ligands that will shift the equilibrium towards the monomeric form of GR, and putatively decrease side effects while conserving the anti-inflammatory action of glucocorticoids, relies on the concept that GRdim acts as a monomer, primarily down-regulating genes through an indirect DNA-binding pathway. Even though in vivo data supports the concept that the A465T mutation (GRdim) is a dimer^15,16^, it has been argued that half-GRE motif recovery in ChIP-seq experiments constitutes strong evidence for monomer GR binding to DNA^33^. In our study, we found that a mostly monomeric GR mutant poorly binds chromatin and has almost no activity, with no evidence of functional half-GRE binding. Moreover, de novo motif analysis shows a high misclassification of half-GREs due to the short sequence length of the motif, making it hard to discriminate against degenerate GREs. We conclude that many of the putative half-sites are in fact degenerate, full GREs. Thus, GRdim does not primarily bind to half-sites. However, when it does, it is unlikely to bind as a monomer at all, but rather as a non-functional dimer.

If the GRdim is dimeric^15^, able to bind DNA (Fig. 2a), and capable of transitioning to a tetramer^19^, why does it not regulate genes efficiently? One of the greatest challenges in the post-genomic era is to functionally link enhancers to the regulation of a specific target gene^42^. Our data shows that GRdim binds ~70% of the sites that wild-type can access, and all are found within pre-accessible chromatin (Fig. 2a). GRdim at these sites can additionally recruit chromatin remodelers such as SMARCA4 and slightly increase accessibility even further, deepening the mystery about which step this mutant cannot perform to activate RNA polymerase II. While we have not been able to pinpoint what those further steps might be, both ChIP-seq and RNA-seq data make apparent this limitation of the GRdim mutant despite its oligomeric status similarity to GRwt^19^. In vitro^43^ and in silico^44^ studies propose that the A465T mutation affects how specific GRE sequences allosterically alter the DNA bound GR structure, which can in principle compromise downstream steps in gene expression regulation. Single-molecule tracking experiments hinted the possibility that GRdim shows faster dynamics than GRwt^45,46^, although the meaning and functional consequences of these differences needs further exploration. As we have previously shown, endogenous GR can act as an initiating factor at GREs that occur within the core particle DNA of a nucleosome^36^. In this sense, another striking possibility is that the ~30% remaining binding sites (Fig. 2a, C1 cluster), i.e. the de novo enhancers, are responsible for most of GR functional activity, consistent with a recent study suggesting only a fraction of GR enhancers have direct glucocorticoid-induced activity^47^. However, explaining the entire GR response solely on these de novo sites seems unlikely because (1) nearby GR-responsive genes are almost as enriched at the shared GRwt/dim sites (Fig. 2b); and (2) expression of a dominant negative AP-1 globally affects GR binding and about half of the GR-regulated genes^39^, but has no effect on the de novo enhancers (Supplementary Fig. 8b). Further investigations are needed to address these questions.

The inability of a dimerization-defective mutant (GRmon) to efficiently bind chromatin and trigger virtually no gene response (Fig. 1c) suggests the monomeric form of GR has a minimal role in GC action and is unlikely to contribute significantly to pharmacology. Hence, the little activity GRdim has is likely to occur through its dimeric form, not as a monomer. Given our genomic and imaging data, we propose that activated GR monomers rarely exist in vivo, and if they exist, they are mainly non-functional. GR appears to be predominantly dimeric once it binds ligand, and upon DNA binding it is most likely a tetramer^28^.

The term *transrepression* refers to a form of tethering wherein the GR negatively modulates the activity of the DNA-bound interacting partner^48^. Paradigmatic examples include AP-1 and NFκB, key factors in inflammatory responses^1^. The proposal that transactivation and transrepression are two distinct, mechanistically separated modes of GR action is exclusively based on the inability of GRdim to bind DNA^10^. While in the current work transrepression hasn’t been directly tested, here we show that GRdim binds chromatin in vivo. This result alone should call into question the reported independence of transrepression from direct DNA binding. In this sense, recent work has indicated the possibility that GR can directly bind AP-1^49^ and NFκB sites^50^. Furthermore, the cross talk between AP-1 and GR networks is much more complex than a simple antagonistic relationship. For example, AP-1 presets the chromatin landscape and facilitates GR binding to accessible regulatory elements genome-wide^39^. While the existence of tethering cannot be ruled out as a viable option for GR-NFκB antagonism^51,52^, alternative pathways were also reported, such as GR inhibiting p65 binding^9,53,54^. In fact, a recent report demonstrated that GR direct DNA binding is essential for both transcriptional activation and repression activities^25^. Given the complexity of potential mechanisms of action, a fully dissociated, independent transactivation vs transrepression model requires revision.

**Fig. 7:**
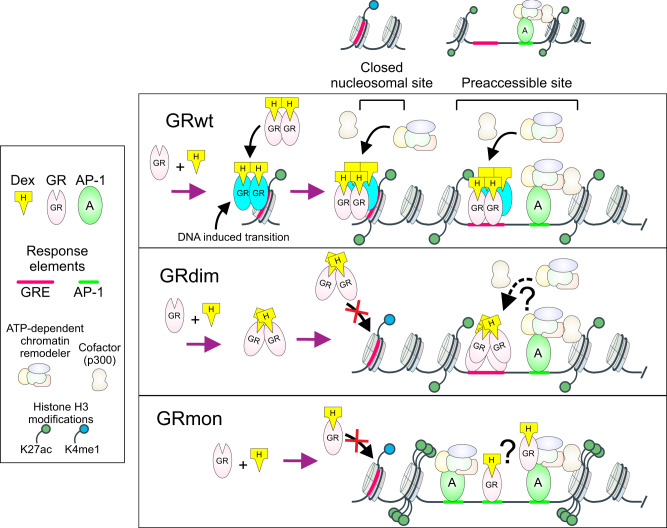
Model of GRwt, GRdim, and GRmon action on chromatin. Binding of liganded GR to chromatin induces a transition from a dimeric to tetrameric state. GRwt can bind to closed nucleosomal sites and to pre-accessible sites. GRwt can recruit chromatin remodelers and other cofactors to both closed and pre-accessible sites to increase chromatin accessibility and influence gene expression. AP-1 (or other initiating factors) maintain chromatin pre-accessible sites prior to receptor binding. GRdim can bind only to pre-accessible sites, where it can recruit chromatin remodelers and other cofactors to a limited extent. GRmon can only bind to strongly pre-accessible sites with high amount of active histone modifications. GRmon can potentially tether to other transcription factors such as AP-1.

Manipulating the oligomeric state of GR between monomers and dimers to gain any pharmacological advantage seems a futile goal. Our data suggests dimerization is necessary but not sufficient to generate an active receptor (results summarized in Fig. 7). In fact, tetrameric GR appears to be the optimal active form of the receptor for both activation and repression of its downstream regulatory gene network^22^. It remains to be seen whether tetramers are the only active DNA-bound form of GR or dimers can also be present at certain enhancers throughout the genome. We propose that the dissociated model of GR function, established primarily by early in vitro studies using the GRdim mutant is no longer consistent with the current literature. Our data and re-analyses of previous genome-wide data indicates GR must form at least a dimer to bind chromatin as measured by ChIP, and that binding does not necessarily lead to efficient gene response. Our genome-wide data, taken together with our microscopy studies, indicate no separation between receptor oligomerization status and whether GR functions as a transcriptional activator or repressor. This has important implications for the therapeutic uses of steroid hormones and the goal of finding selective anti-inflammatory drugs that do not create unwanted side-effects.

## Methods

### Cell culture and generation of cell lines by CRISPR/cas9

All cell lines were grown in Dulbecco’s modified Eagle’s medium (DMEM, Invitrogen) supplemented with 5 μg/ml tetracycline (Sigma–Aldrich #T7660), 10% fetal bovine serum (Gemini), sodium pyruvate, nonessential amino acids, and 2 mM glutamine maintained in a humidifier at 37 C and 5% CO_2_. Cells were plated for experiments in DMEM growth medium supplemented with 10% charcoal/dextran-treated serum for 24 h prior to hormone treatment.

From our previously published GR knock-out (GRKO) cell line^22^, we used CRISPR/Cas9 methods^55^ to reintroduced the GFP-tagged mouse WT, the mouse GR A465T (GRdim) mutation or the mouse GR A465T/I634A (GRmon) double mutation via the GT(Rosa)26Sor locus. The mutant GR sequences were generated using a QuikChange II XL Site-Directed Mutagenesis Kit according to the manufacturer’s instructions (Stratagene). The three GFP-GR containing cell lines (GRwt, GRdim, GRmon) were first selected for their respective GR donor insertion by puromycin and then FACS sorted as a polyclonal population of cells for similar levels of GFP fluorescence and cell-size uniformity. The genome-wide GRwt data were published recently in a study involving a constitutively tetrameric GR mutant shown to act as a “super receptor” with enhanced transcriptional hormone response and chromatin binding^22^. We use the same GRwt data in this study as the standard of comparison to the GRdim and GRmon mutants. All cell lines used in this study are readily available from the authors upon reasonable request.

### RNA Isolation and RNA-seq data analysis

GFP-GR mutant expressing cells were left untreated or treated with 100 nM of Dex for 2 h prior RNA isolation. RNA isolations were performed using the Pure-link RNA kit (Thermo #12183018 A) per the manufacturer’s instructions. RNA-seq libraries were generated from rRNA depleted (Illumina #RS-122-2301) total-RNA samples, using Illumina Stranded Total RNA (Illumina #20020596) according to manufacturer’s instructions. We sequenced at least two biological replicates of each cell line for the untreated condition and three replicates each for the hormone treatment condition using Illumina HiSeq2500 with 126 bp pair-end reads. RTA 2.4.11 was used for Base calling and Bcl2fastq 2.17 was used for demultiplexing allowing 1 mismatch. Trimmomatic 0.36^56^ was used for adapter removal and quality control. RNA-seq alignment to mouse mm10 genome was performed by STAR^57^ using the default parameters with the following modifications: ‘--genomeDir mm10-125 --outSAMunmapped Within --outFilterType BySJout --outFilterMultimapNmax 20 --outFilterMismatchNmax 999 --outFilterMismatchNoverLmax 0.04 --alignIntronMin 20 --alignIntronMax 1000000 --alignMatesGapMax 1000000 --alignSJoverhangMin 8 --limitSjdbInsertNsj 2500000 --alignSJDBoverhangMin 1 --sjdbScore 1 --sjdbFileChrStartEnd mm10-125/sjdbList.out.tab --sjdbGTFfile UCSC_mm10_genes.gtf --peOverlapNbasesMin 10 --alignEndsProtrude 10 ConcordantPair’. All RNA-seq biological replicates correlated well with each other (Supplementary Data 4). Raw count data for a total of 24,940 genes were obtained using analyzeRepeats.pl function in the HOMER pipeline. Low-count genes were removed by requiring more than 15 reads in at least two samples for each gene across the 15 samples, and the remaining 13,465 genes were used for the subsequent analyses. To account for the variability between two batches of experiments and sequencing, one batch for GRwt/GRmon and the other for GRdim, we used the RUVg method in the RUVSeq package^23^ with a minor modification that allows to retain the first and fourth largest singular values for *k* = 2 to estimate unwanted factors in the function. As in silico empirical control genes, we used all 13,465 genes. Factor analysis was performed using the modified RUVg on DESeq2 size factor normalized count data to estimate two factors of unwanted variation; then, the factors were used in the model for DE analysis using DESeq2 to adjust for the unwanted variation^24^. DE genes were identified based on the criteria of a false discovery rate (FDR) cutoff < 0.001 and shrunken log2 fold change (LFC) > 0.6 between no treatment and 2 h hormone treatment. Shrunken LFC was obtained using the adaptive shrinkage estimator^58^ that is available from DESeq2 by ‘ashr’ option. Wald test was used to detect DE genes from the pairwise comparison between Dex treated and control cells with each GR type and test Dex treatment effects that are different across cells with different GR types. Matrix of raw RNA-seq tag counts used in the DESeq2 analysis is provided in Supplementary Data 1. PCA analysis was performed using plotPCA function in EDASeq R library^59^.

### RNA time course and RT-qPCR

GFP-GR mutant expressing cells were left untreated or treated with 100 nM of Dex for 1, 2, 4 or 6 h prior RNA isolation. RNA isolations were performed using the Pure-link RNA kit (Thermo #12183018 A) per the manufacturer’s instructions. cDNA reactions were performed with the NEB Protoscript II kit (#E6560S) per the manufacturer’s instructions. QPCRs were performed with iQ SYBR Green Supermix (Biorad # 1708880) in a Biorad CFX96 machine with duplicate wells for each sample. The sequence of all primers used can be found in Supplementary Table 1.

### ChIP sequencing

The GR mutant expressing cells were grown to ~70% confluence on 150-mm dishes as described above and left untreated or treated with 100 nM of Dex (Sigma–Aldrich) for 1 h. The cells were cross-linked by adding paraformaldehyde (Electron Microscopy Sciences #15710) to a final concentration of 1% (v/v) of the growth medium for 10 min and subsequently quenched with 150 mM glycine for 10 min. The cells were rinsed twice with ice-cold PBS and collected in ice-cold PBS containing Protease Inhibitor Cocktail (Sigma–Aldrich #P2714). After collection, the cell pellets were resuspended in ChIP Lysis Buffer [0.5% (w/v) sodium dodecyl sulfate (SDS), 8 mM EDTA, 40 mM Tris-HCl (pH 8), protease inhibitor cocktail]. Chromatin was sonicated (Bioruptor, Diagenode) to an average DNA length of 200–500 bp and cellular debris was removed by centrifugation. After determining the chromatin concentration, the sample was diluted with ChIP Dilution Buffer [0.01% (w/v) SDS, 1.2 mM EDTA, 16.7 mM Tris-HCl (pH 8), 1.1% (v/v) Triton X-100, 167 mM NaCl, protease inhibitor cocktail] to concentration of 200 μg per ml of chromatin. For immunoprecipitation, 600 ug of chromatin was incubated with antibody (see below for details) coupled onto Dynabeads magnetic beads (Thermo Fisher Scientific #11201D #11203D) with rotation overnight at 4 C. The beads were harvested by magnets and washed once for 10 min in cold room by rotation with 1 ml of ChIP Low Salt Wash Buffer [0.01% (w/v) SDS, 2 mM EDTA, 20 mM Tris-HCl (pH 8), 1% (v/v) Triton X-100, 150 mM NaCl], 1 ml of ChIP High Salt Wash Buffer [0.01% (w/v) SDS, 2 mM EDTA, 20 mM Tris-HCl (pH 8), 1% (v/v) Triton X-100, 500 mM NaCl], and 1 ml of ChIP LiCl Wash Buffer [250 mM LiCl, 1% (w/v) IGEPAL, 1% (w/v) sodium deoxycholate, 10 mM Tris-HCl (pH 8), 1 mM EDTA]. Finally, the beads were washed two times for 2 min in cold room by rotation with 1 ml of TE buffer [1 mM EDTA, 10 mM Tris-HCl (pH 8)]. Antibody-bound chromatin fragments were digested and crosslinks reversed with Reversal Buffer [0.0075% (w/v) SDS, 200 mM NaCl, 10 mM EDTA, 50 mM Tris-HCl (pH 7.5), 25 µg proteinase K (Thermo Fisher Scientific) by incubating at 50 C for 2 h. with shaking and subsequently 7 h at 65 C. ChIP DNA was purified samples with phenol-chloroform extraction and ethanol precipitation with glycogen (Thermo Fisher Scientific) as carrier. ChIP-seq libraries were generated Illumina TruSeq ChIP Sample Prep Kit (Illumina #IP-202-1012) according to manufacturer’s instructions.

### ChIP antibodies

The following antibodies and concentration per immunoprecipitation were used in ChIP. Anti-GR cocktail (3 μg, Santa Cruz #sc-1004; 7.5 μg, Thermo Fisher Scientific #PA1-511A; 15 μg, Thermo Fisher Scientific #BuGR2 MA1-510), anti-GFP (25 μg, Abcam #ab290), anti-H3K27ac (4 μg, Active Motif #39133), anti-H3K4me1 (6 μg, Abcam #ab8895), anti-SMARCA4 (BRG1) (2 μl, Abcam #ab110641).

### ATAC-sequencing

GR mutant expressing cells were grown and hormone treated as done before ChIP sequencing. The cells were detached from the flasks using 5 ml of Accutase (Thermo Fisher Scientific #A6964) by incubating 5 min at RT. Hormone exposed cells were detached with Accutase in the presence of 100 nM Dex. Accutase was inactivated with 7.5 ml of growth media. After collection, the cell pellets were resuspended in a concentration of 6 million cells per ml in Buffer A [15 mM Tris-HCl (pH 8), 15 mM NaCl, 60 mM KCl, 1 mM EDTA, 0.5 mM EGTA, 0.5 mM Spermidine (Sigma–Aldrich #S2626), protease inhibitor cocktail]. Subsequently, equal volume of Buffer A with 0.04% (w/v) IGEPAL (Sigma–Aldrich #I8896) was added, to obtain a concentration of 3 million cells per ml with 0.02% (w/v) IGEPAL and incubated on ice for 10 min, pelleted and washed in Buffer A without IGEPAL. This generated a nuclei preparation with greater than 95% lysed cells as verified by Trypan Blue (Thermo Fisher Scientific #15250061) and counting of nuclei (BioRad TC20). Subsequently, the rest of ATAC followed a published protocol^60^. Briefly, 100,000 nuclei were subjected to Tn5 transposition reaction using 2.5 µl TDE1 from Nextera DNA Library Prep Kit (Illumina #FC-121-1030). After adding the transposition reaction mix, the samples were incubated 30 min at 37 C, and subsequently DNA was purified using MinElute PCR Purification kit (Qiagen #28004). Transposed DNA was PCR amplified using 1.25 μM of Ad1 and 1.25 µM of barcoded Ad2.x (see list below) primers, and NEBNext High-Fidelity 2X PCR Master Mix (New England Biolabs #M0551S). After 5x PCR amplification cycles, appropriate number of additional PCR cycles was determined to retain library complexity. 5 µl aliquot was PCR amplified 20x additional cycles using 0.125 µM of the same primer pair and iQ SYBR Green mix (Bio-Rad #1708882). Additional number of PCR cycles corresponds to one third of the maximum fluorescent intensity in qPCR. After additional PCR cycles, size selection was performed using SPRIselect (Beckman Coulter #B23317) to remove less than 150 bp and more than 800 bp fragments according to manufacturer’s instructions. Size selection was verified using 5% TBE PAGE gels (Bio-Rad #3450049). The index primers for ATAC can be found in Supplementary Table 1.

### ChIP- and ATAC-seq data analysis

Biological duplicate ChIP samples were sequences using Illumina NextSeq 500 with single-end reads, while biological duplicate ATAC samples were sequenced using Illumina HiSeq 4000 with pair-end reads. RTA 2.4.11 was used for Base calling and Bcl2fastq 2.17 was used for demultiplexing allowing 1 mismatch. Trimmomatic 0.36^56^ was used for adapter and quality control. Subsequently the data were aligned to the mouse reference mm10 genome using Bowtie 2^61^ with command Bowtie2 –p 8 –x bowtie2_ref/genome_prefix –U read1.fastq –S result.sam. All ChIP-seq and ATAC-seq biological replicates correlated well with each other (Supplementary Data 4). Subsequent downstream analysis was performed using HOMER^30^. Peaks in each dataset were called using the findPeaks function with style factor for TFs and style histone for histone modifications. GR ChIP from the GRKO cell line was used as control for GR samples while input sample from the GRKO cell lines was used for the other samples. Peak filtering was done with the following parameters; FDR <0.001, >4 FC over control, >4 FC over local background, and >75 tags per site. In addition, untreated GR ChIP sample was used to further filter out noise from GR samples using default parameters on getDifferentialPeaks. DESeq2^24^ through getDiffrentialPeaksReplicates.pl was used to isolate differential binding peaks (FDR < 0.05, FC > 3) between the GR mutants. The same differential binding peaks were also identified with getDifferentialPeaks.pl (Poisson *p*-value < 0.0001, FC > 4).

Log_2_ transformed tag counts were used in scatter plots. Correlation between two samples was determined with Pearson correlation coefficient (PCC). Aggregate plots and heatmaps were generated with 10 bp or 20 bp bins surrounding ±1 kb area around the center of the peak or TSS. All plots were normalized to 10 million mapped reads and further to local tag density, tags per bp per site. Statistical significance in the box plot comparisons was determined with unpaired two-sample t-test. Box plots were represented as Tukey box plot, and n in the comparisons is the number of sites in each cluster. Statistical tests used in the study are commonly used and considered appropriate for the hypotheses tested. The data meet assumptions of population distribution. Variance between the groups that are being statistically compared is similar. AnnotatePeaks.pl was used to calculate the enrichment of sites to different genomic location; promoter, intron, intergenic and miscellaneous (other) sites. Miscellaneous sites consist of UTRs, exons and non-coding RNAs.

De novo motif searches were performed with findMotifsGenome.pl using default parameters (Supplementary Data 2). Pre-defined motif searches were performed with findMotifsGenome.pl using default parameters, and with annotatePeaks.pl. To select the most representative GREs for pre-defined motif searches, we generated GRE aggregate plots (motif per site per bp) with annotatePeaks.pl using of all HOMER NR3C type motifs (gre.motif, gre-raw.motif, are.motif, pgr.motif, pr.motif, ar-half.motif), and CIS-BP^31^ mouse GRE motifs (M05886_2.00, M09318_2.00, M09614_2.00) (Fig. 4a, Supplementary Fig. 7). In addition, we also used half-GRE motifs from CIS-BP (M11124_2.00). PWMs of motifs can be found in Supplementary Data 3. All full SRE HOMER and CIS-BP motifs gave complementary results, especially gre.motif, gre-raw.motif, are.motif, pgr.motif, M05886_2.00, M09318_2.00, and M09614_2.00 motifs. For well-defined GRE, we selected HOMER gre-raw.motif as it is generated from mouse RAW264.7 cells. As degenerate GRE, we selected HOMER pr.motif as it represented the only full SRE motif that compared to others was less stringent in the ACA and TGT core base pairs. As half-GRE, we selected HOMER ar-half.motif as it has been previously used to define GRdim binding to half-GREs^26^. The CIS-BP half-GRE (M11124_2.00) gave complementary results compared to ar-half.motif. After defining the occurrence of well-defined GRE, degenerate GRE, and half-GRE for C1–C4 sites, the percentage of occurrence was displayed as bar graph. For the random sites, we randomly selected 2000 accessible sites using ATAC-seq data generated from Dex-treated GRwt cells^22^. For the random sites in liver, we randomly selected 2000 accessible sites using DNase-seq data generated from mouse liver^38^. Subsequently, we defined for each C1–C4 sites which site harbor only half-GRE, only GRE, both GRE and half-GRE, and no GRE or half-GRE. From the sites that harbored both GRE and half-GRE, we checked potential sequenced that are full GRE but are misclassified as half-GRE. To be considered a misclassification the ACA (or TGT) of the half-GRE was required to overlap with one side of the full GRE. Varying degree of sites that harbored GRE and half-GRE were defined to be misclassified; 885 sites in C1, 1296 sites in C2, 14 sites in C3, and 24 sites in C4. The misclassified sites were moved to only GRE -section. To assess the occurrence of even more degenerate GRE at C3 and C4 sites harboring only half-GRE, custom motifs (AGAnnnTnn, AGAnnnnGn, AGAnnnnnT, AGAnnnTnT, AGAnnnTGn, AGAnnnnGT) were generated using HOMER. Log-odd motif scores were determined with annotatePeaks.pl using HOMER NR3C type motifs (gre.motif, gre-raw.motif, are.motif, pgr.motif, pr.motif, ar-half.motif), or AP1 motif (ap1.motif). Distribution of scores was shown as Tukey box plot with notches depicting the confidence of the median.

Determination of aFOS effect on GR binding at C1–C4 was determined by comparison of normalized GR ChIP-seq tag density in the presence or absence of aFOS. The distance between GR binding sites (C1–C4) and closest AP-1 peak was determined using annotatePeaks.pl. CDF was calculated based on the individual distances.

Association of GR binding sites to Dex-regulated genes (peak-centric analysis) was performed based on linear distance using AnnotatePeaks.pl. C1–C4 cluster sites where checked against the union set of Dex up-regulated genes in GR mutant cells. The data was presented as Tukey box plot with log2 Dex/NT values.

### Immunoblotting

Whole cell extracts (no hormone treatment) for western blots were prepared in RIPA buffer (50 mM Tris pH 8.0, 150 mM NaCl, 1% Tergitol, 0.5% Na-Deoxycholate, 0.1% SDS) with protease inhibitor cocktail (Sigma–Aldrich #P2714) and quantitated by Bradford assay (Biorad #500-006). We ran 30 μg of cell extract on 4-20% PAGE gels (Biorad #4561096) and transferred onto PVDF membranes (Biorad #1704156). Blots were incubated in 5% milk with anti-GR (Santa Cruz #sc-1004) at 1:1000 dilution and GAPDH (Abcam #ab8245) at 1:2000 dilution. For secondary detection, the membranes were probed in 5% milk with HRP-conjugated secondary mouse or rabbit antibodies (#31430 and #31460, respectively; Pierce Thermo Scientific) at 1:2500 dilution. The membranes were incubated with Super Signal Pico detection reagent (Pierce Thermo Scientific #34082 #34083) and visualized using ChemiDoc MP imaging system (Bio-Rad). Full uncropped immunoblot images can be found in Supplementary Fig. 9.

### Reporting summary

Further information on research design is available in the Nature Research Reporting Summary linked to this article.

## Supplementary information

Supplementary Information

Descriptions of Additional Supplementary Files

Supplementary Data 1

Supplementary Data 2

Supplementary Data 3

Supplementary Data 4

Supplementary Data 5

Reporting Summary

## Data Availability

RNA-seq, ChIP-seq data, and ATAC-seq generated for this study were deposited to the NCBI Gene Expression Omnibus (GEO) under accession number GSE117661. Accession numbers for all previously published data used in this study can be found in Supplementary Data 5. TF motifs used in this manuscript can be found at the HOMER database (http://homer.ucsd.edu/homer/) or the CIS-BP database (http://cisbp.ccbr.utoronto.ca). Motif matrixes used in the study can be found in Supplementary Data 3. All other relevant data are available from the corresponding author upon reasonable request. Source data are provided with this paper.

## Source data

Source Data
