# Supplementary material for: Genome-wide binding potential and regulatory activity of the glucocorticoid receptor’s monomeric and dimeric forms
Source: Nat Commun. 2021 Mar 31;12:1987. doi: 10.1038/s41467-021-22234-9 (PMC8012360; doi:10.1038/s41467-021-22234-9)
Supplement: Supplementary file 1 — Supplementary Information [file 41467_2021_22234_MOESM1_ESM.pdf]

Genome-wide Binding Potential and Regulatory Activity of the Glucocorticoid Receptor’s Monomeric and Dimeric Forms

Thomas A. Johnson, Ville Paakinaho, Sohyoung Kim, Gordon L. Hager, and Diego M. Presman

SUPPLEMENTARY INFORMATION

- Supplementary Figures 1-9
- Supplementary Table 1

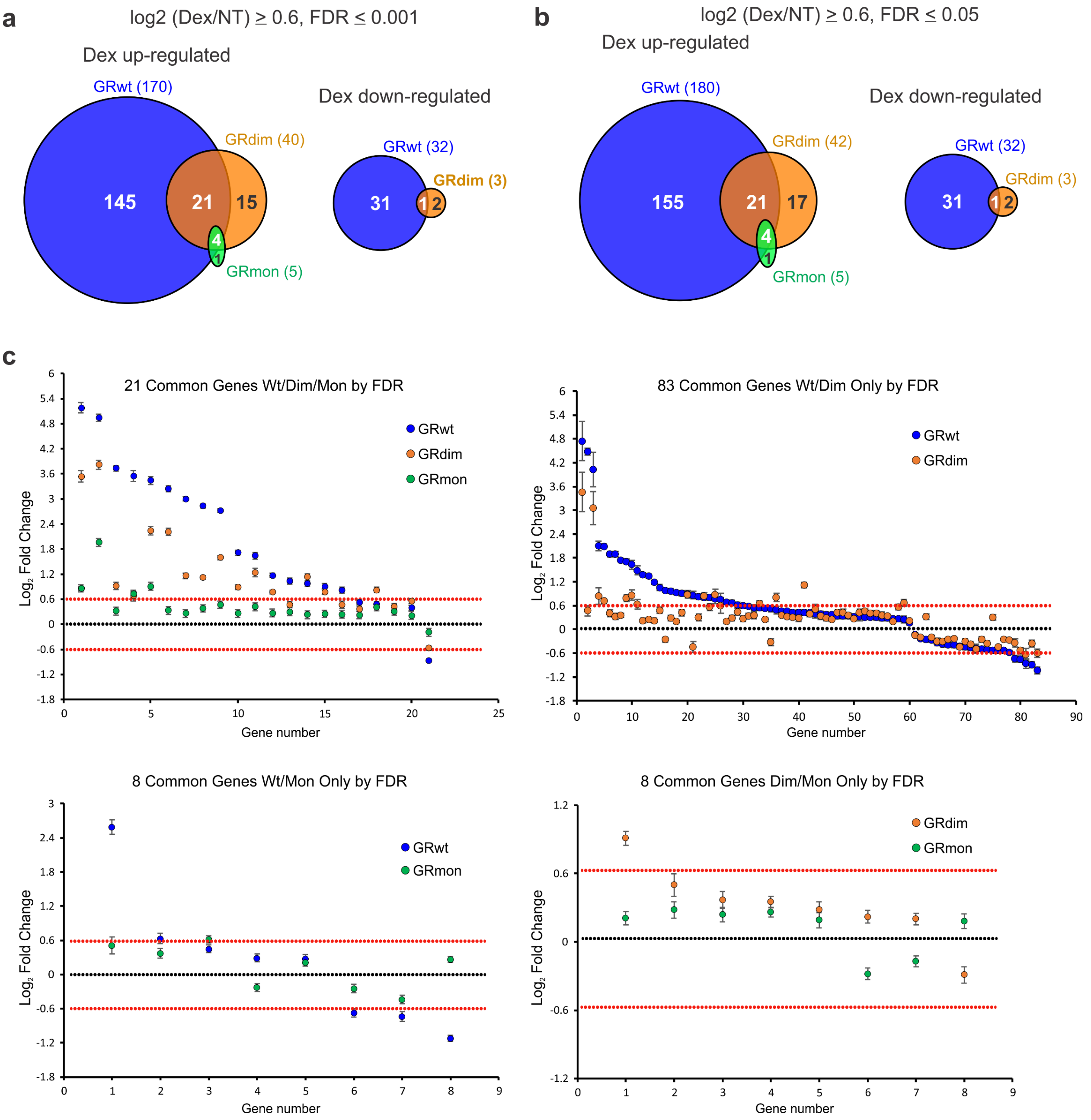

Supplementary Figure 1. **RNA-seq analyses with varying statistical cutoffs.** (a-b) Venn diagrams of up-regulated and down-regulated genes from the RNA-seq data after 2h Dex treatment. FDR and log_2FC as indicated. (c) Scatter plots of shared hormone regulated genes (FDR 0.001; without regard to FC cutoff) between the three GR types, as indicated. Y-axis is log_2 change (Dex/NT) and the red horizontal line denotes $\pm$ log_2 FC = 0.6. NT, non-treated; FDR, false discovery rate; FC, fold change. Error bars are standard deviation of RNA-seq FC replicates.

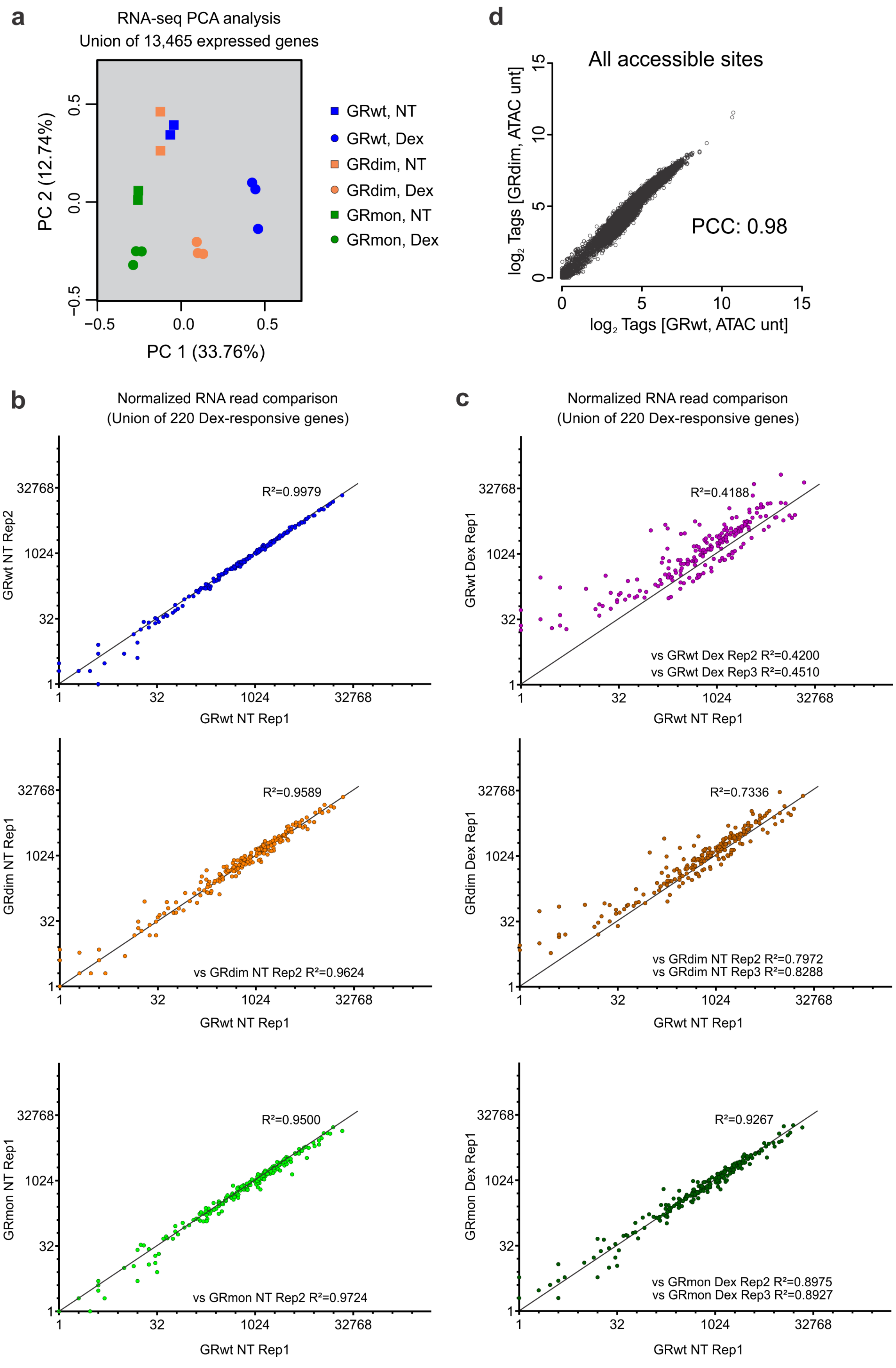
a
RNA-seq PCA analysis
Union of 13,465 expressed genes
PC 2 (12.74%)
PC 1 (33.76%)
GRwt, NT
GRwt, Dex
GRdim, NT
GRdim, Dex
GRmon, NT
GRmon, Dex
d
All accessible sites
log2 Tags [GRdim, ATAC unt]
log2 Tags [GRwt, ATAC unt]
PCC: 0.98
b
Normalized RNA read comparison
(Union of 220 Dex-responsive genes)
GRwt NT Rep2
R²=0.9979
GRwt NT Rep1
GRdim NT Rep1
R²=0.9589
vs GRdim NT Rep2 R²=0.9624
GRmon NT Rep1
R²=0.9500
vs GRmon NT Rep2 R²=0.9724
c
Normalized RNA read comparison
(Union of 220 Dex-responsive genes)
GRwt Dex Rep1
R²=0.4188
vs GRwt Dex Rep2 R²=0.4200
vs GRwt Dex Rep3 R²=0.4510
GRdim Dex Rep1
R²=0.7336
vs GRdim NT Rep2 R²=0.7972
vs GRdim NT Rep3 R²=0.8288
GRmon Dex Rep1
R²=0.9267
vs GRmon Dex Rep2 R²=0.8975
vs GRmon Dex Rep3 R²=0.8927

Supplementary Figure 2. Isogeneity analysis of each mutant cell line based on RNA-seq and accessibility data. (a) Principal component analysis (PCA) of a common set of 13,465 expressed genes (any gene with read counts >15) in the RNA-seq datasets after RUVg normalization (see methods) for GRwt (blue), GRdim (orange), and GRmon (green) samples. Circles denote hormone-treated while squares denote non-treated (NT) condition. The cell lines appear not to be fully isogenic as some variability can be detected between NT conditions. However, the hormone treatment promotes bigger differences respect to NT conditions for GRwt and GRdim. The similarities between GRmon treated and non-treated samples are consistent with the lack of hormone-response for this mutant. (b-c) Scattered plot of the normalized RNA read counts for all dex-regulated genes from the indicated replicate experiments. The scatter plot shows that the variability within biological replicates in GRwt (R^2 = 0.99) is similar to the variability between GRwt and GRdim (R^2 = 0.96) or GRmon (R^2 = 0.95) background levels. On the contrary, there is more variability between the effect of hormone treatment in GRwt (R^2 = 0.42) compared to GRdim (R^2 = 0.73), and GRmon (R^2 = 0.93), reflecting their respective transcriptional capability. (d) Comparison of chromatin accessibility by ATAC-seq in untreated condition between GRwt and GRdim cells. Scatter plot represents normalized $\log_2$ tag counts of all accessible sites. PCC; Pearson correlation coefficient. The comparisons show that the baseline accessibility is similar between cell lines.

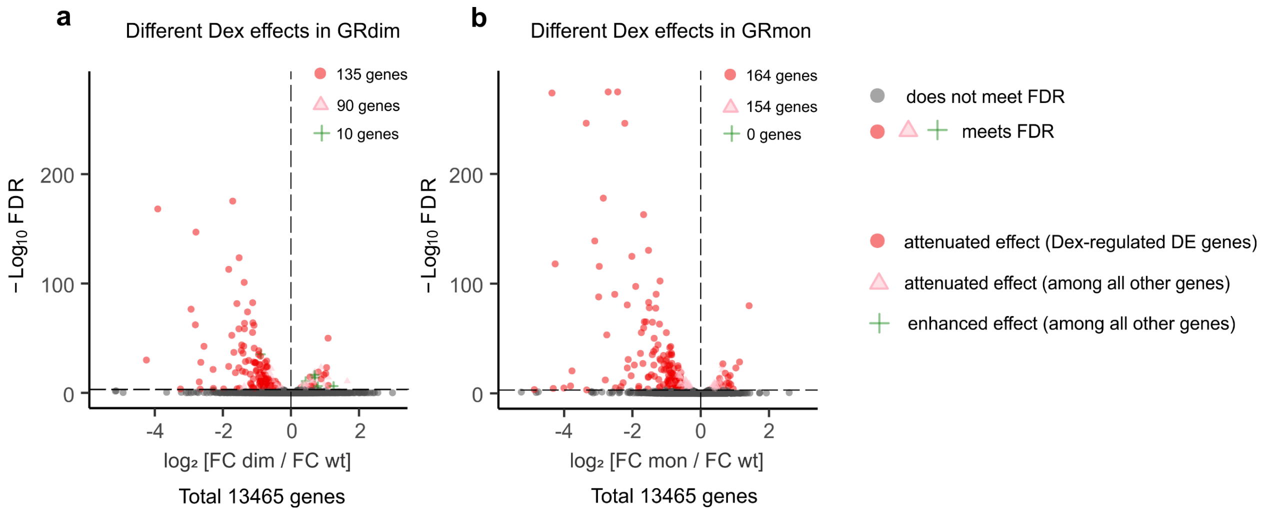

Supplementary Figure 3. Dex-dependent effects across GR types. (**a-b**) To evaluate possible differences in the Dex-response of GRdim and GRmon against GRwt, we evaluated all genes expressed at least in two or more samples (13,465 genes, see methods). Using the Wald test, we statistically assessed how the mutants 'response to Dex compares to GRwt by taking into account the levels of gene expression in untreated cells. The volcano-plots show that 235 genes have a different Dex effect in GRdim (a), while 318 genes show different Dex effects in GRmon (b), compared to the Dex response in GRwt (genes with FDR<0.001, dark-red dots). Among the 235 genes that showed significantly different Dex responses in GRdim, > 95% of genes (225, dark-dots and light-red triangles) present an attenuated response (i.e., smaller fold increase or decrease). Among these 225 genes, 135 genes were hormone-regulated and differentially expressed (DE) genes in GRwt (Fig. 1d). A small fraction of genes (<5%, 10 genes, green cross) shows an enhanced Dex response (i.e., larger fold increase or decrease). Similarly, among the 318 genes

that presents significantly different Dex effects in GRmon, all genes (318) present an attenuated response. Among these 318 genes, 164 were hormone-regulated DE genes in GRwt (Fig. 1d). In conclusion, there is a wide scale attenuated gene induction and suppression in GRdim, and more so in GRmon. FDR, false discovery rate. FC, fold change.

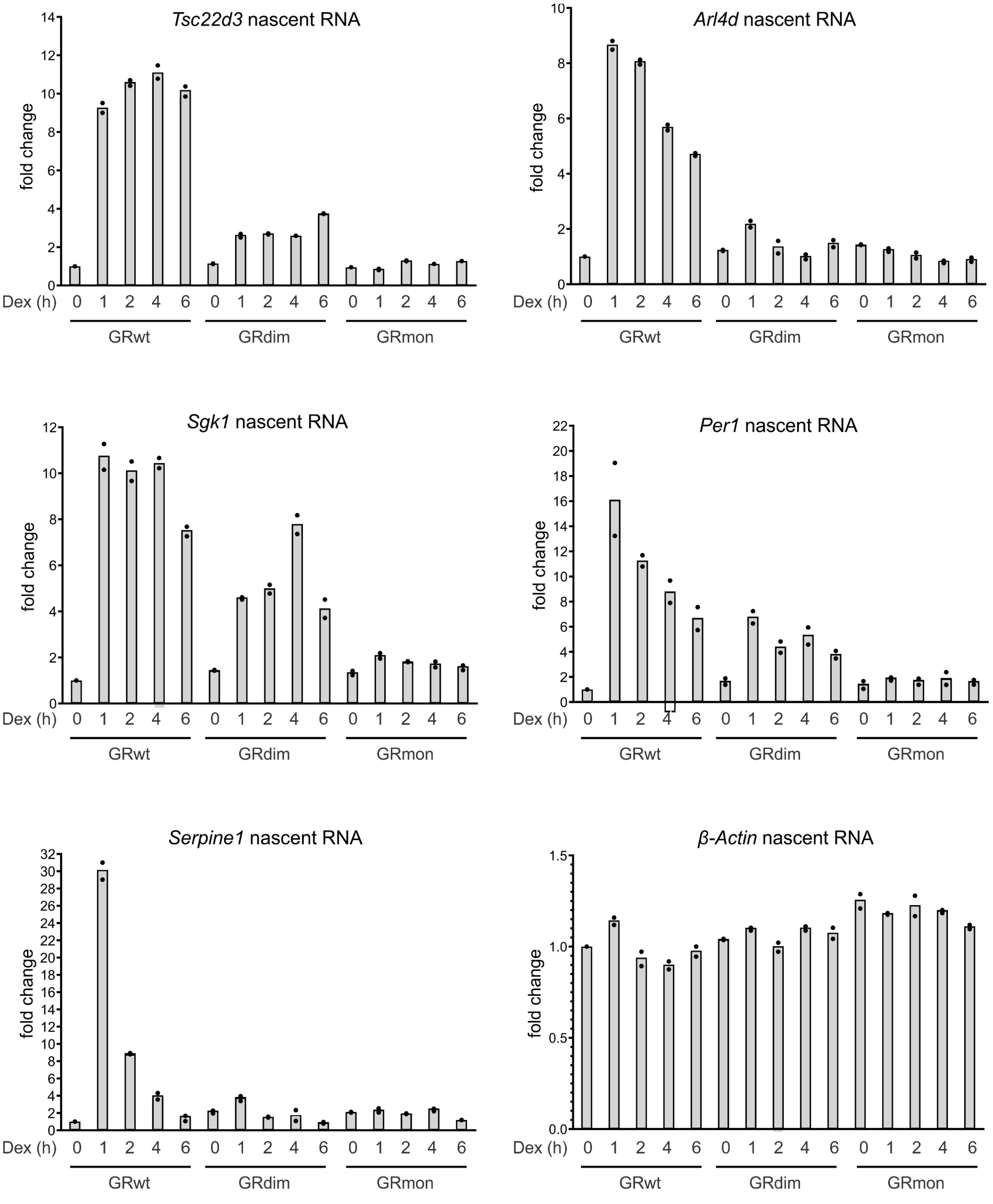

Supplementary Figure 4. **RT-qPCR time course.** RT-qPCR performed on a panel of GR up-regulated genes, with names noted on top of each graph. Cells were left untreated or treated with 100 nM Dex as indicated. Y-axis represents the mean linear fold change compared to the GRwt untreated sample for each gene.

Black dots correspond to the data points for each QPCR replicate (n=2). Source data are provided as a Source Data file.

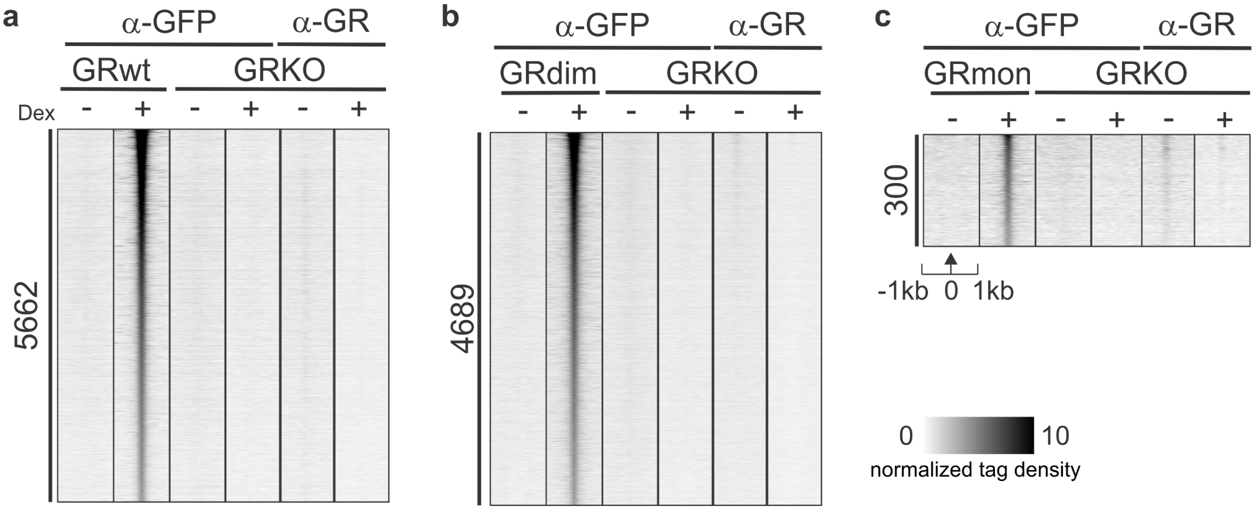

Supplementary Figure 5. GRwt, GRdim, and GRmon cistromes. (a-c) Heat maps comparing GR binding with anti-GR and anti-GFP antibodies at GRKO cells to GRwt (a), GRdim (b), and GRmon (c) binding sites. Each heat map represents ±1kb around the center of the GR peak. The treatment is noted above, and the binding intensity scale is noted below on a linear scale. Heat maps normalized to 10 million reads.

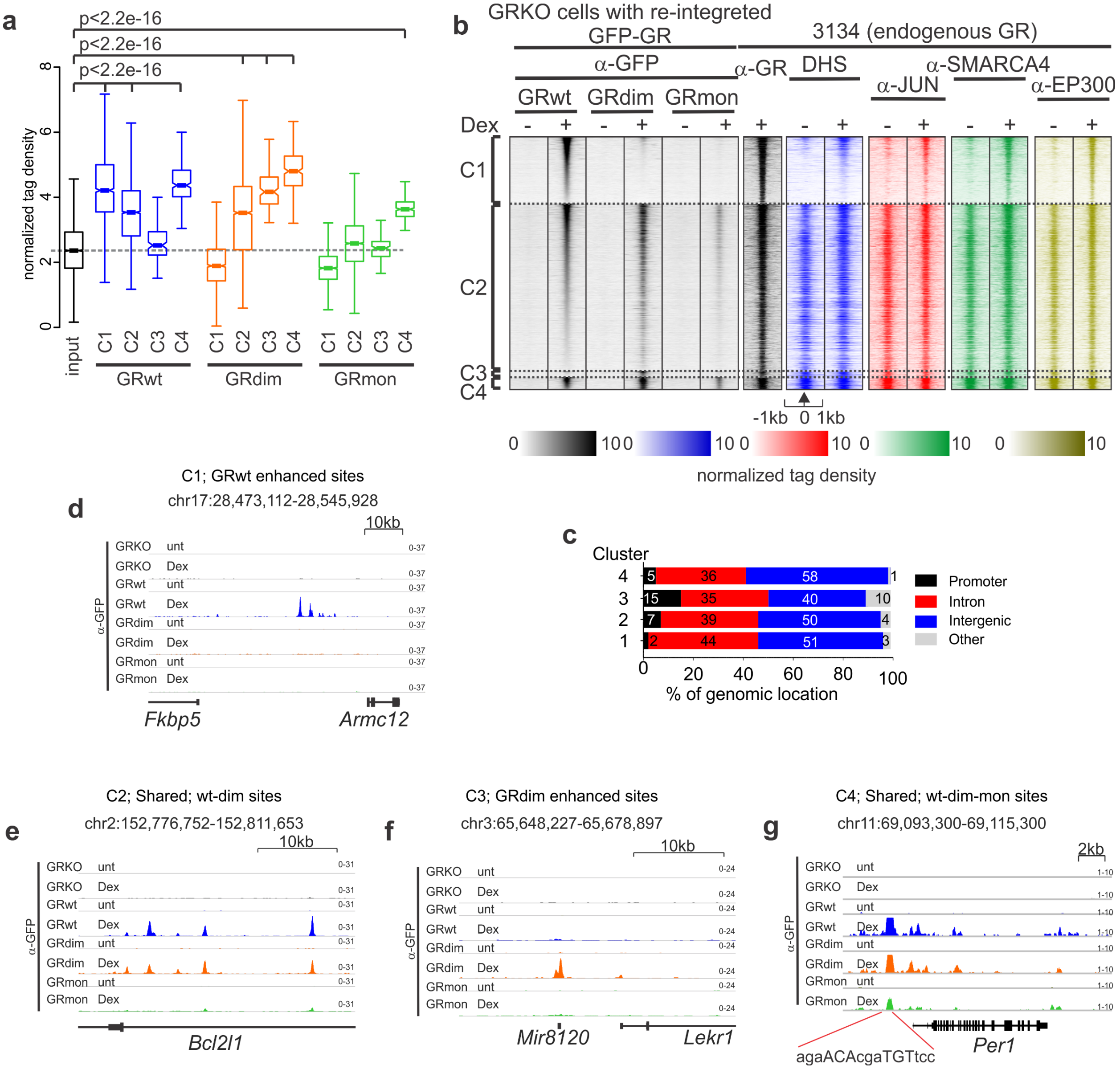

Supplementary Figure 6. Comparison of GRwt, GRdim, and GRmon cistromes. (a) Statistical comparison of GR enrichment to C1-C4 sites. Box plots represent normalized $\log_2$ tag density of input (black), GRwt (blue), GRdim (orange), and GRmon (green) to C1-C4 sites. p-values calculated using unpaired two-sample t-test (two-sided). Box plots normalized to 10 million reads. Box plots were generated with Tukey method with interquartile range (IQR) depicting the 25th, 50th and 75th percentile as box with the median as black bar. Notches depicting the confidence of the median. The whiskers extend 1.5xIQR beyond the box, and outliers depicted as circles extend beyond the 1.5xIQR. C1 n=1609, C2 n=4014, C3 n=144, C4 n=300 sites. (b) Heat maps show data from Fig. 2d alongside GR ChIP-seq; DHS (DNase-seq); JUN, SMARCA4 and EP300 ChIP-seq from parental 3134 cells at C1-C4. Each heat map represents ±1kb around the center of the GR peak. Treatment noted above and binding intensity scale is noted below on a linear scale. Heat maps are sorted based on GRwt binding intensity, except C3 which is sorted based on GRdim binding intensity. Heat

maps are normalized to 10 million reads, and further to local tag density. (c) Bar chart shows percentage of sites at indicated genomic locations for C1-C4 sites. (d-g) Example genome browser tracks of C1 (d), C2 (e), C3 (f) and C4 (g) sites. Genome browser tracks are normalized to 10 million reads. Source data are provided as a Source Data file.

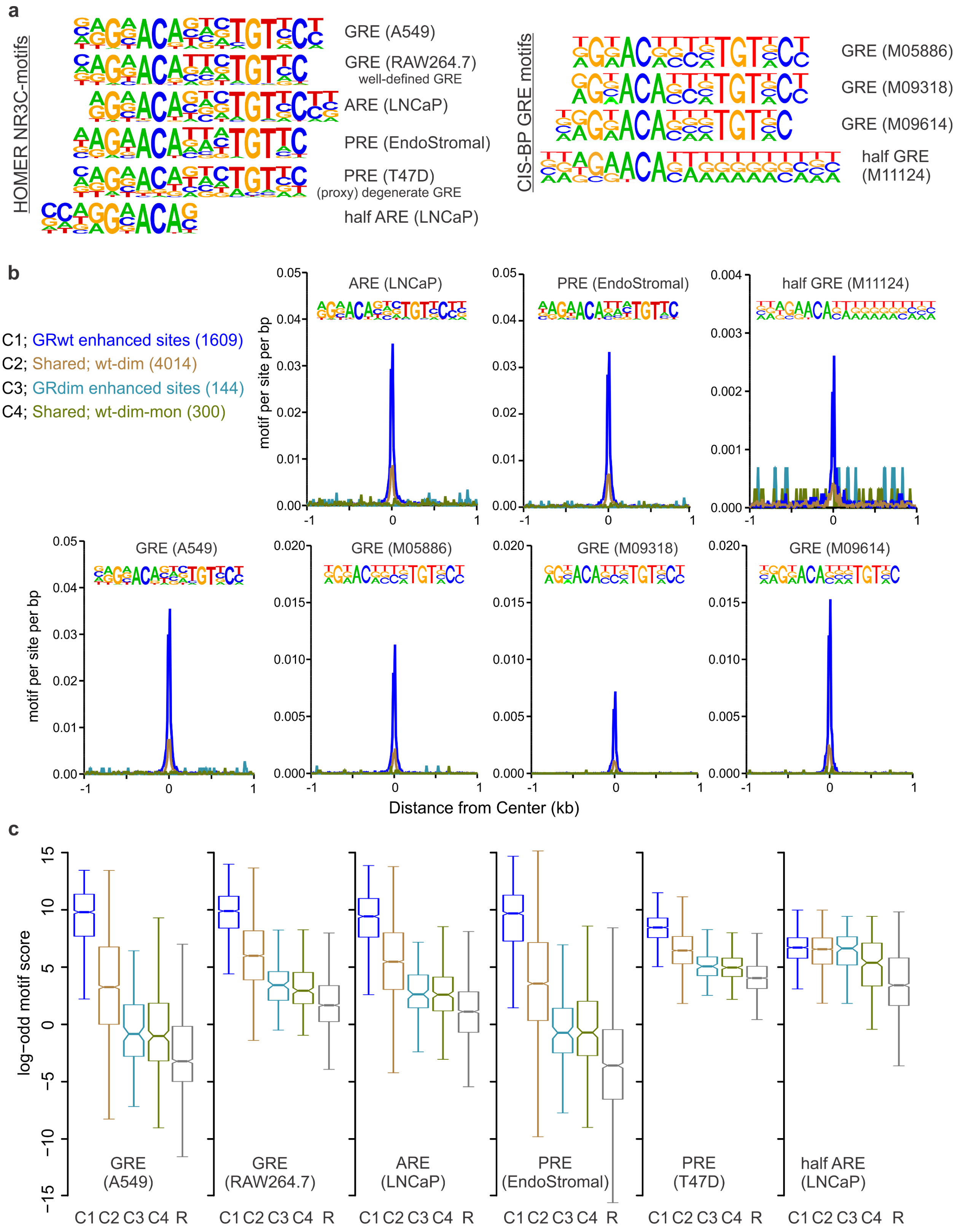

Supplementary Figure 7. Pre-defined motif analyses. (a) Position weight matrix (PWM) logos for NR3C motifs used by HOMER program and mouse GRE motifs by CIS-BP database. Note the more stringent

consensus bases used by CIS-BP motifs. (b) Enrichment of a selected NR3C motifs from HOMER not used in Fig. 4a, and mouse GRE motifs from CIS-BP database at GR ChIP peaks from each cluster (C1-C4). (c) Motif score comparison of NR3C motifs from HOMER at C1 (blue), C2 (gold), C3 (teal), C4 (green) and randomly selected sites (R) (gray). Y-axis shows log-odds motif score. Box plots were generated with Tukey method with interquartile range (IQR) depicting the 25th, 50th and 75th percentile as box with the median as black bar. Notches depicting the confidence of the median. The whiskers extend 1.5xIQR beyond the box, and outliers depicted as circles extend beyond the 1.5xIQR. C1 n=1609, C2 n=4014, C3 n=144, C4 n=300 sites, R n=2000. Source data are provided as a Source Data file.

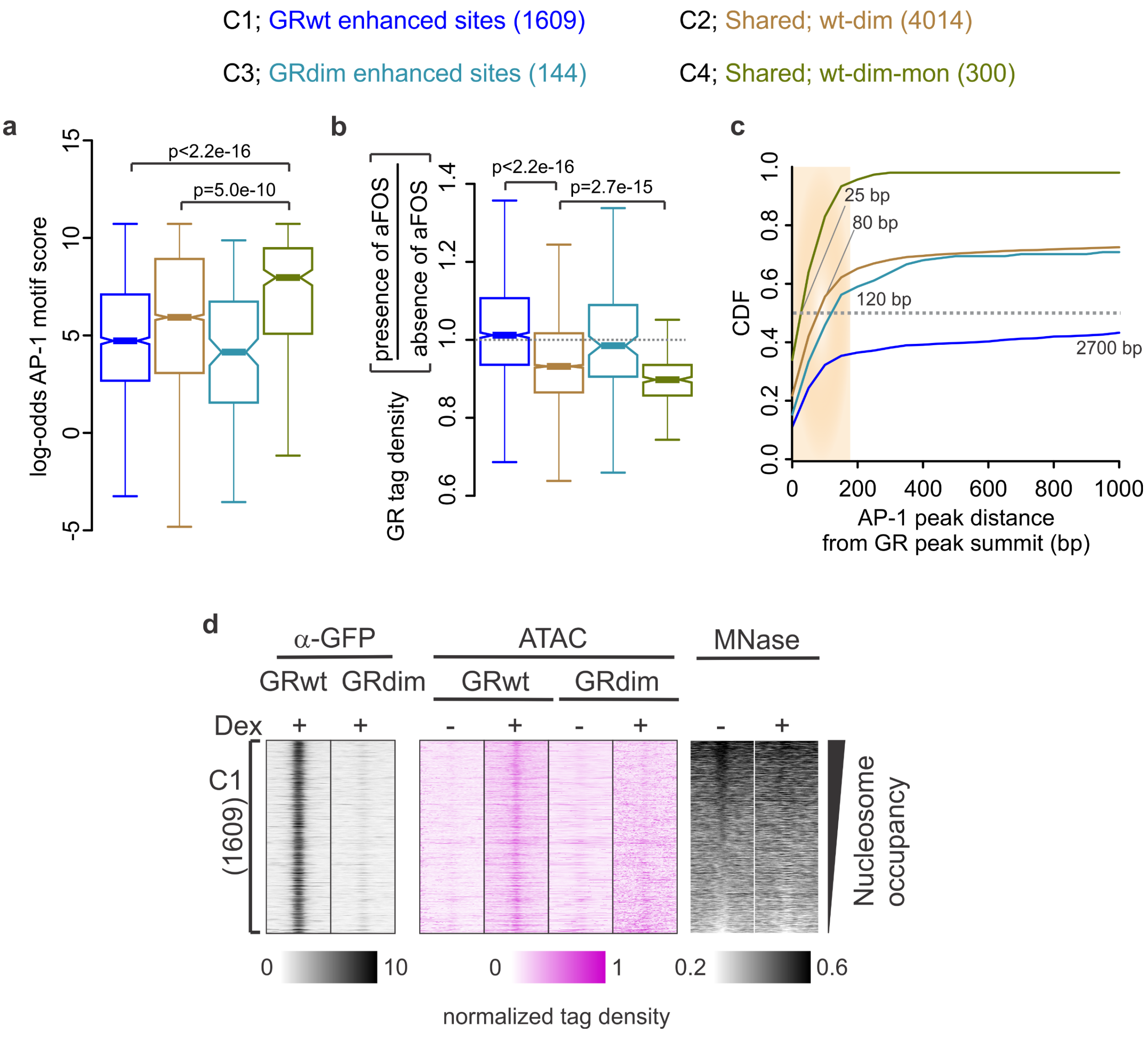

Supplementary Figure 8. GRwt specific sites are independent of initiating factor AP-1 action. (a) Box plots show log-odds motif scores of AP-1 at C1-C4. Color indicates cluster, p-values calculated using unpaired two-sample t-test. (b) Box plots show the effect of dominant negative AP-1, aFOS, for GR binding at C1-C4 sites. Color indicates cluster, p-values calculated using unpaired two-sample t-test (two-sided).

Box plots were generated with Tukey method with interquartile range (IQR) depicting the 25th, 50th and 75th percentile as box with the median as black bar. Notches depicting the confidence of the median. The whiskers extend 1.5xIQR beyond the box, and outliers depicted as circles extend beyond the 1.5xIQR. C1 n=1609, C2 n=4014, C3 n=144, C4 n=300 sites. (c) Cumulative distribution function (CDF) between C1-C4 binding sites and JUN peak in 3134 cells. Each cluster is color-coded with median distance shown for each cluster. Grey dashed line depicts median. (d) Heat maps of GFP ChIP-seq and ATAC-seq from GRwt and GRdim cells, and MNase-seq from 3134 cells in C1 sorted by nucleosome occupancy (based on MNase data) with highest nucleosome occupancy at the top. Each heat map represents ±1kb around the center of the GR peak. Treatment is noted on top and binding intensity scale is noted below on a linear scale. Heat maps normalized to 10 million reads, and further to local tag density. Source data are provided as a Source Data file.

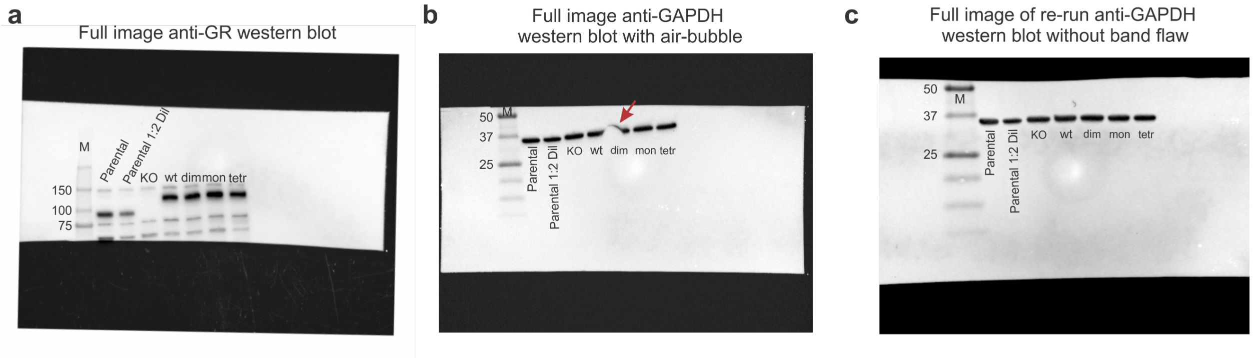

Supplementary Figure 9. Full immunoblots shown in Fig 1b. (a) Immunoblotting using anti-GR (Santa Cruz #sc-1004). (b-c) Immunoblotting against GAPDH (Abcam #ab8245). Due to an air bubble during protein transfer (b, red arrow), the loading control was run again on a different gel (c). M, molecular weight marker (Bio-Rad Precision Plus Protein WesternC Standards).

Supplementary Table 1. Primers used for qPCR experiments and ATAC indexing.

RT-qPCR primers	
Primer	Sequence
Tsc22d3 nascent forward	ACATGATGGTGGCATGAAGA
Tsc22d3 nascent reverse	TCTTCTCAAGCAGCTCACGA
Arl4d nascent forward	AAAAGGAATATTTAGAAGGGTAGGGTGC
Arl4d nascent reverse	GCCATTTCAGTCAAGTGGTTCCC
Sgk nascent#2 forward	GAAACAGAGAAGGATGGGCCTGAAC
Sgk nascent#2 reverse	GATCTCAGCTCCAGCACCACCAC
Per1 nascent forward	CTTCTGGCAATGGCAAGGACTC
Per1 nascent reverse	CAGCATCATGCCATCATACACAC
Serpine1 nascent forward	CCGAGAGCTTTGTGAAGGAG
Serpine1 nascent reverse	GAGGGTGAGAGATGGAGACG
beta-actin forward	GCTGGAAAAGAGCCTCAGGGC
beta-actin reverse	CGCATCCTCTTCCTCCCTGGAG

ATAC index primers	
Primer	Sequence
Ad1	AATGATACGGCGACCACCGAGATCTACACTCGTCGGCAGCGTCAGATGTG
Ad2.1	CAAGCAGAAGACGGCATACGAGATTCGCCTTAGTCTCGTGGGCTCGGAGATGT
Ad2.2	CAAGCAGAAGACGGCATACGAGATCTAGTACGGTCTCGTGGGCTCGGAGATGT
Ad2.3	CAAGCAGAAGACGGCATACGAGATTTCTGCCTGTCTCGTGGGCTCGGAGATGT
Ad2.4	CAAGCAGAAGACGGCATACGAGATGCTCAGGAGTCTCGTGGGCTCGGAGATGT
Ad2.5	CAAGCAGAAGACGGCATACGAGATAGGAGTCCGTCTCGTGGGCTCGGAGATGT
Ad2.6	CAAGCAGAAGACGGCATACGAGATCATGCCTAGTCTCGTGGGCTCGGAGATGT
Ad2.7	CAAGCAGAAGACGGCATACGAGATGTAGAGAGGTCTCGTGGGCTCGGAGATGT
Ad2.8	CAAGCAGAAGACGGCATACGAGATCCTCTCTGGTCTCGTGGGCTCGGAGATGT
