# Supplementary material for: Genome-wide binding potential and regulatory activity of the glucocorticoid receptor’s monomeric and dimeric forms
Source: Nat Commun. 2021 Mar 31;12:1987. doi: 10.1038/s41467-021-22234-9 (PMC8012360; doi:10.1038/s41467-021-22234-9)
Supplement: Supplementary file 2 — Descriptions of Additional Supplementary Files [file 41467_2021_22234_MOESM2_ESM.pdf]

Description of Additional Supplementary Files

Supplementary Data 1. RNA-seq data

Supplementary Data 2. HOMER enriched motifs

Supplementary Data 3. Motifs used in the pre-defined searches

Supplementary Data 4. Pearson's correlation coefficient between RNA-seq samples

Supplementary Data 5. Previously published genome-wide datasets used in this work
