# Supplementary material for: Genome-wide binding potential and regulatory activity of the glucocorticoid receptor’s monomeric and dimeric forms
Source: Nat Commun. 2021 Mar 31;12:1987. doi: 10.1038/s41467-021-22234-9 (PMC8012360; doi:10.1038/s41467-021-22234-9)
Supplement: Supplementary file 8 — Reporting Summary [file 41467_2021_22234_MOESM8_ESM.pdf]

natureresearch

Corresponding author(s): Gordon L. Hager, Diego M. Presman

Last updated by author(s): Feb 5, 2021

Reporting Summary

Nature Research wishes to improve the reproducibility of the work that we publish. This form provides structure for consistency and transparency in reporting. For further information on Nature Research policies, see Authors & Referees and the Editorial Policy Checklist .

Statistics

For all statistical analyses, confirm that the following items are present in the figure legend, table legend, main text, or Methods section.

n/a	Confirmed	
☐	☒	The exact sample size (*n*) for each experimental group/condition, given as a discrete number and unit of measurement
☐	☒	A statement on whether measurements were taken from distinct samples or whether the same sample was measured repeatedly
☐	☒	The statistical test(s) used AND whether they are one- or two-sided<br>*Only common tests should be described solely by name; describe more complex techniques in the Methods section.*
☒	☐	A description of all covariates tested
☐	☒	A description of any assumptions or corrections, such as tests of normality and adjustment for multiple comparisons
☐	☒	A full description of the statistical parameters including central tendency (e.g. means) or other basic estimates (e.g. regression coefficient) AND variation (e.g. standard deviation) or associated estimates of uncertainty (e.g. confidence intervals)
☐	☒	For null hypothesis testing, the test statistic (e.g. *F*, *t*, *r*) with confidence intervals, effect sizes, degrees of freedom and *P* value noted<br>*Give P values as exact values whenever suitable.*
☒	☐	For Bayesian analysis, information on the choice of priors and Markov chain Monte Carlo settings
☒	☐	For hierarchical and complex designs, identification of the appropriate level for tests and full reporting of outcomes
☐	☒	Estimates of effect sizes (e.g. Cohen's *d*, Pearson's *r*), indicating how they were calculated

Our web collection on statistics for biologists contains articles on many of the points above.

Software and code

Policy information about availability of computer code

Data collection

The following software was used in the study:
RTA 2.4.11
Bcl2fastq 2.17
Trimmomatic 0.36
STAR 2.7
Bowtie2 2.3
HOMER 4.9
R 3.5.2
R libraries:
DESeq2 1.28.1
RUVSeq 1.22.0
ashr 2.2.47
EDASeq 2.22.0

Data analysis

RNA-seq data analysis:
RTA (v2.4.11) used for Base calling and Bcl2fastq (v2.17) used for de-multiplexing allowing 1 mismatch. Trimmomatic (v0.36) used for adapter removal and quality control. Alignment by STAR (v2.7) with default parameters with the following modifications: '--genomeDir mm10-125 --outSAMunmapped Within --outFilterType BySJout --outFilterMultimapNmax 20 --outFilterMismatchNmax 999 --outFilterMismatchNoverLmax 0.04 --alignIntronMin 20 --alignIntronMax 1000000 --alignMatesGapMax 1000000 --alignSJoverhangMin 8 --limitSjdbInsertNsj 2500000 --alignSJDBoverhangMin 1 --sjdbScore 1 --sjdbFileChrStartEnd mm10-125/sjdbList.out.tab --sjdbGTFfile UCSC_mm10_genes.gtf --peOverlapNbasesMin 10 --alignEndsProtrude 10 ConcordantPair'. Downstream analysis performed using HOMER (v4.9) by the analyzeRepeats.pl function, and RUVg (v1.22.0) (k=2, the 1st and 4th largest singular values), DESeq2 (v1.28.1) with ashr (v2.2.47) shrinkage. Dex-regulated genes had FDR <0.001 and shrunken FC of 1.5 (log2 +/- 0.6). Principal component analysis performed using R package (v3.5.2) EDASeq (v2.22.0) with default settings.

ChIP-seq and ATAC-seq data analysis:
RTA (v2.4.11) used for Base calling and Bcl2fastq (v2.17) used for de-multiplexing allowing 1 mismatch. Trimmomatic (v0.36) used for adapter removal and quality control. Alignment by Bowtie2 (v2.3) with command Bowtie2 –p 8 –x bowtie2_ref/genome_prefix –U read1.fastq –S result.sam. Downstream analysis performed using HOMER (v4.9). Peak calling; FDR<0.001, FC>4 over control, FC>4 over local background, and tag per site>75. GR ChIP or input sample from the GRKO cell line used as control. Differential binding peaks between the GR mutants had FDR<0.05, FC>3. Correlation between two samples determined with Pearson correlation coefficient (PCC). Aggregate plots and heatmaps generated using AnnotatePeaks.pl. AnnotatePeaks.pl used to calculate the enrichment of sites to different genomic location. De novo motif searches performed with findMotifsGenome.pl using default parameters. Pre-defined motif searches performed with findMotifsGenome.pl using default parameters, and with annotatePeaks.pl. PWMs used in the pre-defined motif searches are provided in Supplementary Table 5. Association of GR binding sites to Dex-regulated genes performed using AnnotatePeaks.pl.

For manuscripts utilizing custom algorithms or software that are central to the research but not yet described in published literature, software must be made available to editors/reviewers. We strongly encourage code deposition in a community repository (e.g. GitHub). See the Nature Research guidelines for submitting code & software for further information.

Data

Policy information about availability of data

All manuscripts must include a data availability statement. This statement should provide the following information, where applicable:

- Accession codes, unique identifiers, or web links for publicly available datasets
- A list of figures that have associated raw data
- A description of any restrictions on data availability

RNA-seq, ChIP-seq data, and ATAC-seq generated for this study were deposited to the NCBI Gene Expression Omnibus (GEO; https://www.ncbi.nlm. nih.gov/geo/) under accession number GSE117661. Accession numbers for all previously published data used in this study can be found in Supplementary Data 5. Source data are provided with this paper, including processed data underlying Figs. 2b; 3a-d; 4a; 5b; 6a-f; and Supplementary Figs. 4; 6a; 7b-c; 8a-b. TF motifs used in this manuscript can be found at the HOMER database (http://homer.ucsd.edu/homer/) or the CIS-BP database (http://cisbp.ccbr.utoronto.ca). Motif matrixes used in the study can be found in Supplementary Data 3. All other relevant data are available from the corresponding author upon reasonable request.

Field-specific reporting

Please select the one below that is the best fit for your research. If you are not sure, read the appropriate sections before making your selection.

[x] Life sciences   [ ] Behavioural & social sciences   [ ] Ecological, evolutionary & environmental sciences

For a reference copy of the document with all sections, see nature.com/documents/nr-reporting-summary-flat.pdf

Life sciences study design

All studies must disclose on these points even when the disclosure is negative.

Sample size	Sample size was chosen based on ENCODE guidelines, which requires at least 2 biological independent replicates for genome-wide analysis (see Landt et el, Genome Res 2012). No statistical methods were used to predetermine sample size.
Data exclusions	When applicable, positive and negative controls were used in each experiment. If these controls failed, the data was excluded.
Replication	In general, all experiments were generated from 2-3 biological replicate samples. Biological replicate samples were generated independent of each other. All attempts at replication were successful.
Randomization	Randomization was not used in this study due to the use of cell lines
Blinding	Investigators were not blinded as this was not relevant to the analysis of the data generated here, and the same pipelines and scripts were used to analyze all samples.

Reporting for specific materials, systems and methods

We require information from authors about some types of materials, experimental systems and methods used in many studies. Here, indicate whether each material, system or method listed is relevant to your study. If you are not sure if a list item applies to your research, read the appropriate section before selecting a response.

Materials & experimental systems

n/a	Involved in the study	
	x	Antibodies
	x	Eukaryotic cell lines
x		Palaeontology
x		Animals and other organisms
x		Human research participants
x		Clinical data

Methods

n/a	Involved in the study	
	x	ChIP-seq
x		Flow cytometry
x		MRI-based neuroimaging

Antibodies

Antibodies used	For ChIP-seq:<br>Anti-GR cocktail: 3 ug, Santa Cruz #sc-1004; 7.5 ug, Thermo Fisher #PA1-511A; 15 ug, Thermo Fisher #MA1-510<br>Anti-GFP: 25 ug, Abcam #ab290<br>Anti-H3K27ac: 4 ug, Active Motif #39133<br>Anti-H3K4me1: 6 ug, Abcam #ab8895<br>Anti-SMARCA4: 2 ul, Abcam #ab110641<br><br>For Immunoblotting:<br>Primary anti-GR: Santa Cruz #sc-1004 at 1:1000 dilution<br>Primary anti-GAPDH: Abcam #ab8245 at 1:2000 dilution<br>Secondary anti-mouse: Pierce Thermo # 31430 at 1:2500 dilution<br>Secondary anti-rabbit: Pierce Thermo #31460 at 1:2500 dilution
Validation	Anti-GR cocktail: Validated for ChIP-seq in-house (John et al, Nat Gen 2011) in mouse cell lines.<br><br>Anti-GFP: Validated for WB and IP in human cell lines on the manufacturer's website and for ChIP-seq in-house in mouse cell lines (Paakinaho et al, Gen Res 2019).<br><br>Anti-H3K27ac: Validated for ChIP-seq on the manufacturer's website from human to yeast and for ChIP-seq in-house in mouse cell lines (Paakinaho et al, Gen Res 2019).<br><br>Anti-H3K4me1: Validated for ChIP on the manufacturer's website in human cell lines and for ChIP-seq in-house in mouse cell lines (Paakinaho et al, Gen Res 2019).<br><br>Anti-SMARCA4: Validated for WB and IP in mouse cell lines on the manufacturer's website. Validated for ChIP-seq in-house (Paakinaho et al, Gen Res 2019).<br><br>Anti-GR: Validated for WB in mouse cell lines on the manufacturer's website.<br><br>Anti-GAPDH: Validated for WB in mouse cell lines on the manufacturer's website.

Eukaryotic cell lines

Policy information about cell lines

Cell line source(s)	Cell lines utilized in the study are derived from C127 (ATCC; CRL-1804). 3134 and 3617 parental cell lines have been generated in the Hager lab. Generation of GRKO and GFP-GR expressing cell lines are described in Materials and Methods section and at Paakinaho et al. 2019 Genome Res
Authentication	The cell lines utilized harbor MMTV array (McNally et al. 2000 Science). The formation of the MMTV array is routinely used as authentication of the cells
Mycoplasma contamination	Mycoplasma contamination was checked annually. Results were negative
Commonly misidentified lines<br>(See ICLAC register)	No commonly misidentified cell lines were utilized in the study.

ChIP-seq

Data deposition

- [x] Confirm that both raw and final processed data have been deposited in a public database such as GEO.
- [x] Confirm that you have deposited or provided access to graph files (e.g. BED files) for the called peaks.

Data access links<br>*May remain private before publication.*	https://www.ncbi.nlm.nih.gov/geo/query/acc.cgi?acc=GSE117661<br><br>GRKO and GRwt ChIP-seq samples utilized in the study can be accessed at<br>https://www.ncbi.nlm.nih.gov/geo/query/acc.cgi?acc=GSE108634
Files in database submission	ChIPseq-GRKO-GFP-GRdim_abGFP_NT_rep1<br>ChIPseq-GRKO-GFP-GRdim_abGFP_NT_rep2<br>ChIPseq-GRKO-GFP-GRdim_abGFP_DEX_rep1<br>ChIPseq-GRKO-GFP-GRdim_abGFP_DEX_rep2<br>ChIPseq-GRKO-GFP-GRdim_abH3K27ac_NT_rep1<br>ChIPseq-GRKO-GFP-GRdim_abH3K27ac_NT_rep2<br>ChIPseq-GRKO-GFP-GRdim_abH3K27ac_DEX_rep1<br>ChIPseq-GRKO-GFP-GRdim_abH3K27ac_DEX_rep2<br>ChIPseq-GRKO-GFP-GRdim_abH3K4me1_NT_rep1<br>ChIPseq-GRKO-GFP-GRdim_abH3K4me1_NT_rep2<br>ChIPseq-GRKO-GFP-GRdim_abH3K4me1_DEX_rep1<br>ChIPseq-GRKO-GFP-GRdim_abH3K4me1_DEX_rep2<br>ChIPseq-GRKO-GFP-GRdim_abSMARCA4_NT_rep1<br>ChIPseq-GRKO-GFP-GRdim_abSMARCA4_DEX_rep1<br>ChIPseq-GRKO-GFP-GRmon_abGFP_NT_rep1<br>ChIPseq-GRKO-GFP-GRmon_abGFP_NT_rep2<br>ChIPseq-GRKO-GFP-GRmon_abGFP_DEX_rep1<br>ChIPseq-GRKO-GFP-GRmon_abGFP_DEX_rep2
Genome browser session<br>(e.g. UCSC)	No longer applicable

Methodology

Replicates	In general, ChIP-seq experiments were generated from 2 biological replicate samples. Replicate concordant was assessed by Pearson correlation coefficiency (PCC). PCC values for applicable samples is provided in Supplementary Data 4.
Sequencing depth	Sequencing depth (i.e. number of unique mapped reads) is provided for each sample in Supplementary Data 4.
Antibodies	All antibodies that have been used for ChIP-seq are described in Materials and Methods section and in GEO
Peak calling parameters	Peaks were called using HOMER program version 4.9 with findPeaks with the following parameters; FDR<0.001 (0.1%), FC>4 over control, FC>4 over local background, tags per site>75
Data quality	All peaks identified with above described peak calling are identified with parameters FC>5 and FDR<0.05 (5%).<br>The number of replicate concordant GR peaks detected with our original peak calling:<br>GRwt Dex rep1&2, 5 923; GRdim Dex rep1&2, 4 458; GRmon Dex rep1&2, 300.<br>The number of GR peaks detected from each replicate sample with FDR<5% and FC>5 peak calling:<br>GRwt Dex rep1, 6 964; GRwt Dex rep2, 9 068; GRdim Dex rep1, 6 768; GRdim Dex rep2, 7 317; GRmon Dex rep1, 2 205; GRmon Dex rep2, 2 984.
Software	RTA 2.4.11 was used for Base calling and Bcl2fastq 2.17 was used for demultiplexing allowing 1 mismatch. Trimmomatic 0.36 was used for adapter and quality control. The data were aligned to the mouse reference mm10 genome using Bowtie2 with command Bowtie2 –p 8 –x bowtie2_ref/genome_prefix –U read1.fastq –S result.sam. Downstream analysis was performed using HOMER version 4.9. Peaks in each dataset were called using the findPeaks function with style factor for TFs and style histone for histone modifications. GR ChIP from the GRKO cell line was used as control for GR samples while input sample from the GRKO cell lines was used for the other samples. Peak filtering was done as indicates above at "Peak calling parameters". Details on further analyses are provided in the Methods section.
